# Advances in the Rapid Diagnostic of Viral Respiratory Tract Infections

**DOI:** 10.3389/fcimb.2022.807253

**Published:** 2022-02-10

**Authors:** Gratiela Gradisteanu Pircalabioru, Florina Silvia Iliescu, Grigore Mihaescu, Alina Irina Cucu, Octavian Narcis Ionescu, Melania Popescu, Monica Simion, Liliana Burlibasa, Mihaela Tica, Mariana Carmen Chifiriuc, Ciprian Iliescu

**Affiliations:** ^1^ Research Institute of the University of Bucharest, Bucharest, Romania; ^2^ National Institute for Research and Development in Microtechnologies—IMT, Bucharest, Romania; ^3^ Faculty of Biology, University of Bucharest, Bucharest, Romania; ^4^ Petroleum-Gas University of Ploiesti, Ploiesti, Romania; ^5^ Emergency University Hospital, Bucharest, Romania; ^6^ Academy of Romanian Scientists, Bucharest, Romania; ^7^ The Romanian Academy, Bucharest, Romania; ^8^ Faculty of Applied Chemistry and Materials Science, University “Politehnica” of Bucharest, Bucharest, Romania

**Keywords:** point-of-care, microfluidics, biosensors, viral respiratory infection, IoT - internet of things

## Abstract

Viral infections are a significant public health problem, primarily due to their high transmission rate, various pathological manifestations, ranging from mild to severe symptoms and subclinical onset. Laboratory diagnostic tests for infectious diseases, with a short enough turnaround time, are promising tools to improve patient care, antiviral therapeutic decisions, and infection prevention. Numerous microbiological molecular and serological diagnostic testing devices have been developed and authorised as benchtop systems, and only a few as rapid miniaturised, fully automated, portable digital platforms. Their successful implementation in virology relies on their performance and impact on patient management. This review describes the current progress and perspectives in developing micro- and nanotechnology-based solutions for rapidly detecting human viral respiratory infectious diseases. It provides a nonexhaustive overview of currently commercially available and under-study diagnostic testing methods and discusses the sampling and viral genetic trends as preanalytical components influencing the results. We describe the clinical performance of tests, focusing on alternatives such as microfluidics-, biosensors-, Internet-of-Things (IoT)-based devices for rapid and accurate viral loads and immunological responses detection. The conclusions highlight the potential impact of the newly developed devices on laboratory diagnostic and clinical outcomes.

## Introduction

The increasing incidence of acute respiratory tract infections (RTI) leads to high mortality in children and adults worldwide. The RTI account for 56 million deaths in 2019 in all age groups representing the third worldwide leading cause of death after cardiovascular diseases and neoplasms (IHME, 2021). The current protocols used to confirm the diagnosis of viral infection rely on virologic laboratory methods that either isolate and identify the pathogens or monitor the levels of antibodies in the body fluids (Loeffelholz and Tang, 2020; Lu et al., 2021). These methods involve laboratory techniques that require specialised equipment and technical expertise. Also, the preanalytical components (e.g., samples collection, transportation, preparation for batch testing, etc.) contributes to a slow turnaround time that further delays the results and related diagnostic and therapeutic decisions. Correctly identifying the viral aetiology of respiratory tract infectious diseases remains challenging despite the availability of multiplex nucleic acid amplification tests (NAATs) due to the problematic interpretation of the results. Moreover, essential deviations from the ideal scenario of acute infection progressing to pathogens’ clearance, such as persistence, latency, reactivation, late disease, and drug resistance, influence the pattern for a particular virus, the immune competence of the host, and the decision about ordering tests and interpreting the results (Booss and Tselis, 2014).

Recently, microfluidics developed to be integrated into rapid and specific diagnostic tools. Lab-on-a-chip (LOC) technologies evolved from single-task-based analysis into advanced integrated systems for complex work (Schumacher et al., 2012). The complexity resides in the multiple interconnected elements such as microchannels, valves, mixers, pumps, chambers for reaction and detection assembled on the same platform where each microfluidic component performs distinct operations for specific and elaborated laboratory protocols. Benchtop protocols comprising tasks such as reagent storage, fluid transport, fluid mixing, product detection, and collection can be finalised directly on the self-operated microfluidic device, (Jung et al., 2015; Park et al., 2020) or near the patient bedside as point-of-care testing (POCT) (Wang et al., 2020). One of the clinical advantages resides in the ability of LOC-based techniques to be developed for viral detection (Aalberts et al., 2012). Previous work demonstrated the potential of microfluidic devices for single virus diagnoses platforms that included sample preparation and detection of Ebola (Coarsey et al., 2019), dengue fever -DENV (Darwish et al., 2018), hepatitis (Duchesne and Lacombe, 2018), human immunodeficiency virus -HIV- Mauk et al., 2017), highly pathogenic avian influenza viruses -HPAIVs- (Liu et al., 2018) or for testing the presence of multiple pathogens (Chen et al., 2021). The new strategies provide quick microbiological analysis for correct differential diagnosis, effective treatment, and disease clearance without complications. To reduce the risk of RTI complications, POCT can be a valuable instrument, especially in developing countries (WHO, 2020). These methods will decrease not only the empirical administration of antibiotics, the risk of selecting drug-resistant strains, or the rate of developing large-scale outbreaks, but eventually the healthcare costs.

Recent reviews approached the impact of microfluidics and biosensing on the rapid detection of RTI. For instance, an overview of available technologies used to detect SARS-CoV-2 in clinical laboratories was presented by Safiabadi and colleagues (Safiabadi et al., 2021). In 2014, Anema and colleagues suggested the potential use of digital surveillance for public health emergencies of international concern, such as the Ebola virus (Anema et al., 2014). Liu and colleagues approached the challenges of automated sample preparation, amplification and signal transduction for rapid diagnosis (Liu et al., 2021). Furthermore, the key advantages in the selection of smart material-based POCT platforms were reviewed by Sow and colleagues (Sow et al., 2020), while the “metal-organic frameworks” (metal clusters + organic linkers) involved in the viral detection (NAAT and immunological) were analysed by Wang and colleagues (Wang et al., 2020). Work focused on technology for virologic diagnostic and highlighted NAAT technologies for rapid molecular diagnostic (Lee et al., 2019) and the influence of micro- and nanotechnology on viral diagnostic (Nasrollahi et al., 2021). The present review covers the advances in microbiological diagnostic of viral RTI, focusing on miniaturised systems and evaluating the clinical perspectives for further use as POCT. We provide a nonexhaustive overview of conventional viral detection and infection monitoring methods and technological improvements. We discuss the potential of immunoassays and nucleic acid (NA) amplification and the new approaches such as microfluidics and biosensors-based techniques as rapid diagnostic platforms for viral respiratory infections detection methods and monitoring. Since viral infections impose stringent detection and spread monitoring, we present the emerging Internet-of-Things (IoT) and highlight their potential as a future solution in the virology diagnostic and respiratory infections prophylaxis.

## Trends in Viral Respiratory Infections Etiology

Besides influenza and respiratory syncytial viruses (RSV) responsible for the highest mortality and hospitalisation rates, other viral pathogens such as parainfluenza, corona-, adeno-, boca-, and rhinoviruses are known for leading to high morbidity (severe diseases in addition to mild upper respiratory tract infections), mortality, and economic burden (Fendrick et al., 2003; Azar and Landry, 2018). Most respiratory infections are mainly caused by viruses and are often mild and self-limited. However, the zoonotic viruses with tropism for the human respiratory tract such as severe acute respiratory syndrome coronavirus (SARS-CoV, 2003), influenza A (H1N1, 2009), Middle East respiratory syndrome coronavirus (MERS-CoV, 2012), and severe acute respiratory syndrome coronavirus 2 (SARS-CoV-2, 2019) identified over the past decades affected the lower respiratory tract fast and infected millions of humans (Peeri et al., 2020). Since the natural progression of viral infections in humans depends on the virulence of viral strains, the general health, immune status and reaction of the hosts, the clinical manifestations range from mild to lethal acute or chronic debilitating complications (Basile et al., 2018; Jin et al., 2021). Moreover, it has been acknowledged that some respiratory infections viruses are drug-resistant strains, e.g., cytomegalovirus isolated from immunocompromised patients, human adenovirus 14p1, group C rhinovirus, human metapneumovirus (hMPV), polyomaviruses-KIPyV, WUPyV- (Ma et al., 2021). Furthermore, the zoonotic agents (avian influenza viruses and MERS-CoV- (Assiri et al., 2013) and severe acute respiratory syndrome -SARS coronaviruses- (Ksiazek et al., 2003) with extensive genetic modifications through recombinant mutations (coronaviruses), gene reassortment (avian influenza viruses), or cascade mutations, exceeded the species barrier and amplified the risk of epidemic and pandemic emerging viral infections with the interhuman transmission (Dhama et al., 2020; Rodriguez-Morales et al., 2020).

Since the viral respiratory tract infections resemble other similar infections of bacterial origins, the differential diagnosis and adequate isolation and treatment measures are complex. For these reasons, the World Health Organization has included diagnosis and diagnostic tests for severe acute respiratory infections into a critical research agenda to develop effective laboratory techniques, from sampling to viral diagnosis and enable early and affordable detection and monitoring of the viruses that play critical roles in pandemics (WHO, 2020).

## Sample Collection Methods for Respiratory Viral Infections

Non-invasive and invasive sampling methods have been used to isolate respiratory viruses in cell cultures and detect antigens in immunoassays. Currently, collecting respiratory ciliated epithelial cells or cell-free viruses is from swabs, aspirates, washes, brushes, lavages, or aspirates at various respiratory trach (RT) levels. The reference method is the nasopharyngeal swab collected by the healthcare personnel (Larios et al., 2011). The biological origin of clinical samples and time since recovery from patients significantly impact the accurate and early detection of viruses (Macfarlane et al., 2005; Lessler et al., 2009). The choice is based on the specificity and sensitivity of the diagnostic testing and the segment of the respiratory tract affected, either upper, (Su et al., 2016) or lower (Zaki et al., 2012), the viruses targeted (Hui et al., 2016) or the age group of patients (Doan et al., 2009). For instance, viruses accumulate at various locations within the respiratory tract during incubation times (Ahluwalia et al., 1987), and sputum may not be productive to be a valid sampling method indicating the aspirate collection (Lessler et al., 2009).

Furthermore, invasive respiratory samples collection such as nasopharyngeal aspirates or swabs are stressful, especially in children and if repeated testing is required. For these reasons, adequate virologic testing includes various sampling methods and sources. The clinical samples to diagnose respiratory infections are collected from various segments of the the respiratory tract or from the bloodstream. The methods are invasive from lavages swabs, aspirates from the anterior nasal, nasopharyngeal, oropharyngeal, bronchoalveolar samples, to phlebotomy for whole blood or serum. Since most of the techniques are invasive, anterior nare swabs or facial tissues were suggested as mitigators (Blaschke et al., 2011). SARS CoV-2 nucleic acid is generally detectable in saliva specimens during the acute phase of infection (Azzi et al., 2020).

Upper airway sampling is relatively simple, can be performed at the bedside, and is minimally invasive. Other specimens may be used instead of blood to detect different viral upper RTI, including SARS-CoV2 (Khan et al., 2017; Wyllie et al., 2020). In this sense, various respiratory samples such as oropharyngeal swabs/throat swabs (OPS/TS), nasopharyngeal swabs (NPS) or nasopharyngeal aspirates (NPA) are employed for the diagnosis of viral RTI (Loens et al., 2009; Corstjens et al., 2016; Charlton et al., 2019). The nasopharyngeal and nasal swabs (flocked, rayon, polyurethane) performed in conjunction with the present diagnostic assays equivalated the traditionally used nasal wash and aspirates specimens (Daley et al., 2006; Chan et al., 2008; Scansen et al., 2010). Furthermore, combined nose and throat specimens contributed to an excellent sensitivity of the polymerase chain reaction (PCR)-based test for influenza virus and RSV (Lambert et al., 2008). The collection protocols may also be altered to increase efficiency while maintaining sensitivity. For instance, parent-collected specimens could be used for PCR testing with equivalent sensitivity to swabs collected by the healthcare professionals in pediatrics (Lambert et al., 2008). The United States Centres for Disease Control and Prevention (CDC) currently offers and demonstrates the guidelines for each procedure while respecting the manufacturers’ recommended specimen types. The CDC recommends collecting the upper respiratory NP swab. Collection of an OP specimen is a lower priority and, if collected, should be combined in the same tube as the NP swab. (CDC, 2021a) Food and Drug Administration (FDA)-cleared or validated laboratory tests (Centres for Disease Control and Prevention, Specimen collection, 2021). Recently, diagnostic testing from NPS and OPS have been approved by FDA to be used on commercial platforms and laboratory-developed tests (Thwe and Ren, 2021). The sampling methods give different detection sensitivities, depending on the respiratory virus. In this sense, Charlton et al. offer practical guidance about selecting specimen type, appropriate sampling time and detection technique concerning various factors, such as the clinical presentation, patient age, the nature of the potential pathogen (Charlton et al., 2019). Aside from the specimen mentioned above, salivary tests have been suggested as an alternative to OPS and NPS (Robinson et al., 2008). It has been demonstrated that human saliva contains specific antibodies for a wide range of viruses multiplying in the respiratory tract: SARS-CoV (Liu et al., 2011) SARS-CoV-2, CMV, Dengue, Ebola, enteroviruses, EBV, HSV 1, 2, influenza A, mumps, measles, poliovirus, rabies, rhino-, rubella, polyoma (BKV, JCV, WUV, KIV), and hepatitis (VHA, VHB, VHC) viruses (Corstjens et al., 2016; Baghizadeh, 2020). Therefore, self-collected specimens may become promising non-invasive diagnostic specimens (To et al., 2020). Newly developed methods based on viral agents in the exhaled breath to complement the existing sampling techniques and monitor the patients for airborne diseases (Rahmani et al., 2020; Soto et al., 2021). Moreover, wearable collectors have the potential to be integrated into a POCT system, like the one demonstrated by Rombach and colleagues (Rombach et al., 2020). For the most sensitive detection of viruses, the collection and testing of upper and lower respiratory samples such as sputum or bronchoalveolar lavage fluid (BAL) are recommended (Cheng et al., 2004). In contrast, lower airway sampling in patients with complex or severe disease (e.g., in intensive care, immune-compromised) or in children with chronic or recurrent respiratory tract symptoms, invasive, requiring a bronchoalveolar lavage (BAL) (De Blic et al., 2000). However, bronchoscopy increases the risk of healthcare workers contamination through aerosol droplets created during the procedure. Therefore, donning proper personal protective equipment (PPE) is crucial. One meta-analysis provided evidence to support NPW, MTS and NPS from 16 sampling methods as the ones with higher diagnostic values for viral respiratory infections. The study also highlighted that each sampling method presents advantages and disadvantages. Therefore, selecting a particular sampling method is influenced by the pathophysiology and pathogenesis of each outbreak. For instance, positive rates, less comfort, and cost supported MTS, while sputum provided higher detection rate for coronaviruses, such as SARS-CoV-2 (Hou et al., 2020). Furthermore, the protocols consider that viral pneumonia cases do not produce purulent sputum and recommend bronchoscopy in the early stages or repeated NPS/NPW to increase the success rate. Interestingly, although generally less reliable than respiratory specimens, the stool, urine, and blood samples contain SARS-CoV and MERS-CoV RNA, and SARS-CoV RNA is consistently detected in faeces at about two weeks after symptom onset (Cheng et al., 2004; Xu et al., 2005).

## Established Diagnostic Methods of Viral Infections

The virologic diagnostic of respiratory infections usually relies on hospital-based procedures to facilitate epidemiological surveillance (Doherty et al., 1998), implementation of timely antiviral therapy (Rocholl et al., 2004), control of nosocomial infections (Groothuis et al., 2008), and wise management of resources (Benito-Fernández et al., 2006) and antibiotics (Doan et al., 2009). Currently, monitoring the respiratory viral infections intends to:

1. measure the plasma levels of antibodies (IgG and IgM) in response to viruses, and
2. detect the viral load in the respiratory specimens.

The quantitative and qualitative analysis reflects the functionality of the host immune system and indicates viral replication in the primary infection site (Lu et al., 2021).

The classical methods to diagnose viral infections are represented by cell culture for the virus isolation (reference method) (Leland and Ginocchio, 2007) and the serological methods for identification (Loeffelholz and Chonmaitree, 2010). Virus isolation is generally performed on sensitive cell cultures inoculated with a sample of tissue or fluid collected from patients to allow the viral multiplication (Leland and Ginocchio, 2007). However, culture-based methods are time-consuming, expensive (depending on equipment and trained operators), and therefore performed mainly in specialised laboratories. The immunoassay testing measures the immunological responses to the virus to differentiate between exposed asymptomatic, acutely, or mildly sick, and recovered cases. The serological diagnosis is a systematic clinical approach to detect respiratory viruses and includes *serotyping* (detection of viral antigens in the patients’ serum) and *serodiagnosis* (detection and quantification of specific antibodies in patients’ bodily fluids). **Table 1** in **Supplementary Table 1** presents a brief comparison of these methods.

The immunoassays detect viral antigens or specific antibodies with high sensitivity and are routine methods for protein detection, especially at significantly low levels, as in influenza’s case (Khanna et al., 2001). Compared to the rapid antibody detection tests, which are qualitative and do not discriminate between recent and old infections (Khalaf et al., 2020), the quantitative assays for specific antibodies (IgM, IgG and IgA) identify the viral agent and follow up the dynamics of the antiviral specific immune response, to establish the infection stages and discriminate between acute and chronic infections (Corstjens et al., 2016). In the case of SARS-CoV infection, the specific antibodies occur 10-20 days after symptoms appear, (Yeh et al., 2014) and the immunological profile shows false-negative results during the window between the viral infection and the start of antibody production (Loeffelholz and Tang, 2020). Since the window for rapid tests is narrow, testing will make with higher sensitivity methods such as PCR and reverse transcriptase (RT)-PCR, loop-mediated amplification (LAMP), and strand displacement amplification, which amplify a specific sequence of the viral genome. These methods, known as nucleic acid amplification techniques (NAAT), inactivate the virions during preliminary purification steps (Zhang et al., 2020) and can simultaneously detect multiple viruses (Lu et al., 2021). However, the presence of nucleic acid does not always mean active infection (Babiker et al., 2021), and significant issues stem during results interpretation: a weak signal explaining either the end of an infection or a recent evolving one and undetectable virus in the upper respiratory tract samples in the respiratory tract infections (Crozier et al., 2021). Furthermore, since respiratory viruses are prone to antigenic drift due to genetic point mutations and reassortment, it is fundamental to identify these changes within the viral genomes. The exciting advantage compared to conventional methods is diagnosing severe pneumonia, detecting coinfections in severe pneumonia patients or those with unknown origin infections. Furthermore, developing high‐throughput whole-genome sequencing (WGS) portable platforms is crucial in identifying viral transmission better than subgenomic sequences (Zhang et al., 2020). Therefore, the evolving next‐generation sequencing (NGS) technologies provide cost-effective rapid sequencing of exomes, transcriptomes, and genomes with increasing potential for diagnosing and identifying respiratory pathogens (Mercer and Salit, 2021). **Table 2** **(** **Supplementary Table 2** **)** presents non-exhaustively the specific diagnostic testing for viral respiratory infections, either as single or multiplex diagnostic testing.

## Microfluidics for Virologic Testing

Since the first miniaturised microfluidic device was developed in 1970 at Stanford University, (Terry et al., 1979) the technologies in microfluidics and LOC (Weibel et al., 2007) advanced towards automation and the use of smaller volumes of clinical samples for a virus purification as efficient as the standard protocols. Microfluidic systems were developed as portable point-of-care devices or “Doctor’s office at home” to detect pathogens and diagnose infectious diseases (Nasseri et al., 2018). Zenhausern and colleagues reviewed microfluidic sample preparation methods such as bead-based, droplet-based, structure-based, fluid-properties-based and described the principles that allow these portable devices to extract, purify and concentrate the viral samples for biological analysis (Zenhausern et al., 2021). Microfluidic-based detection employs nucleic acids amplification, blood chemistry assays, flow cytometry and immunoassays (Li et al., 2021). The techniques differ in terms of turnaround time and cost-effectiveness (e.g., the quantity and number of reagents) the reproducibility. For instance, immunoassays use a simple strip, are rapid and simple diagnostic platforms, compared with blood chemistry testing, which is complex (e.g., measure tens of physiological parameters in one go), and time-consuming. Nucleic acid amplification techniques, however, require a limited copy number of nucleic acids for diagnosis. Furthermore, the micro-assays provide rapid, highly accurate results, as user-friendly and cheap devices (Whitesides, 2006) that perform specimen preparation, reagent manipulation, bioreaction and virus detection on the same miniaturised platform (at very low concentrations and in small sample volumes). The devices incorporate micro-channels and chambers (10-100 µm) designed to match the intended application based on the physical and biological properties of the targeted micro-organisms. The crucial steps are: (1) the design of the microchannels, (2) the manufacture of the integrated platform (soft lithography), and (3) the quantification of the chemicals and biomarkers involved in the process. LOC platforms have common elements with microarrays and biosensors (e.g., the substrates materials), and specific ones such as polymers (e.g., polytetrafluoroethylene, polymethylmethacrylate- (Becker and Gärtner, 2008) or biopolymers (e.g., calcium alginate, cross-linked gelatin) for the skeleton (Ertl et al., 2004). Detection chips on silicon substrates ensure the specificity of biological analysis (Morens et al., 2004). Furthermore, high detection ability of the devices (sensitivity and specificity) imposes a combination of several types of equipment for data acquisition, signal processing, amplification, and monitoring. However, attentive microenvironment control, mainly temperature and mechanical stress, is crucial for the desired functionality. The complexity of the device and the high sensitivity and specificity will ensure the real-time analysis specific to point-of-care-testing (POCT) (Lagally et al., 2000).

## Point-of-Care (POC) Testing Devices

### Generalities

The major problem of the RTI diagnosis is the lack of standardised, rapid, and accurate testing for a differential diagnosis: accurate identification of viruses, bacteria, and fungi, followed by their drug-sensitivity or -resistance profiling. Consequently, in the absence of appropriate and timely therapeutic and epidemiological measures, the detrimental impact of a viral infection increases exponentially (Burbelo et al., 2019). For instance, in influenza A virus infection, treatment with neuraminidase inhibitors (NAIs) should be started within 48 hours after the onset of symptoms. Therefore, it is imperative to create simple, cheap methodological alternatives as POCT. The POCT devices must meet the following criteria: accessibility, availability, sensitivity, specificity, user-friendliness, speed, no accessory equipment required (Drain et al., 2014; Iliescu et al., 2021). The purpose of using POCT is to get a quick diagnosis after analysis of bodily fluids (e.g., blood, serum, urine, or saliva) samples and to provide the clinicians with valuable and timely information for the optimal therapeutic decision (Nelson et al., 2020).

### Types of POCT Used in the Diagnosis of Viral Infections

The use of POCT for viruses was hampered by the low sensitivity of antigen detection tests and the limited spectrum of detected viruses (Brendish et al., 2015), but this field of research has significantly advanced in the last years mainly due to the COVID-19 pandemic. POC tests, molecular or non-molecular, detect different biomarkers, including antibodies, whose concentration in the body fluids changes during the infectious process (O’Sullivan et al., 2019). POC tests, especially non-molecular tests, have several advantages. For instance, they do not require permanent dedicated space or expensive laboratory equipment, clinical laboratory facilities or surgical expertise; they provide real-time results; the infection can be detected in an early stage, and this facilitates rapid therapeutic interventions, eliminating unnecessary investigations.

The point of care diagnostics market is segmented into cardiometabolic testing, glucose monitoring, pregnancy and fertility testing, haematology testing, coagulation testing, infectious disease testing, tumour/cancer marker testing, cholesterol testing, urinalysis testing, and other POC products. The infectious diseases segment is divided into influenza testing, HIV testing, sexually transmitted disease testing, hepatitis C testing, tropical disease testing, respiratory infection testing, other infectious disease testing and healthcare-associated infection testing. Due to the COVID-19 pandemic, which leads to a growing demand for rapid test kits for faster diagnosis at public places, the POC diagnostics market has encountered substantial growth. The POC diagnostics market was US$ 36,000.4 million in 2021 and is expected to reach US$ 82,958.3 million by 2028 (Business Wire, 2021).

*Molecular POCT methods* for multiple detections of respiratory viruses are based on the identification of one or more nucleic acid sequences (RNA or DNA) specific for the pathogen after amplification using a PCR-based method. The PCR based techniques need between 20 and 100 minutes (involving reverse transcription for RNA detection) for achieving 30-40 amplification cycles at 72°C. The isothermal amplification of nucleic acids using LAMP (Zhang et al., 2019) is a new variant of DNA amplification, using a DNA-polymerase acting at a constant temperature of 60-65°C, thus eliminating the need for a thermocycler and being cheaper and easier to run) (Drancourt et al., 2016).

A new generation of molecular technologies for POC diagnosis is CRISPR (clustered regularly interspaced short palindromic repeats). The CRISPR - POC was implemented after discovering that certain enzymes of this system, namely Cas13 (CRISPR associated) have RNase activity related to various RNA molecules. The RNase activity of CRISPR enzymes is used for signal amplification and nucleic acids detection. The first step is described as the isothermal amplification of the DNA and RT-RNA in the sample to increase the amount of viral RNA as potential targets of RNase. Subsequently, the Cas13a enzyme with specific activity to the specific viral RNA sequence is added. If viral RNA is present, Cas13a binds to it and is activated to cleave a fluorescent-labelled reporter molecule, which produces a light signal measured on a fluorescent plate. Through these steps results, the presence of RNA is detected in a few hours (Burbelo et al., 2019; Li et al., 2019).

The CRISPR/Cas12a-based precision technique, called SARS-CoV-2 DNA endonuclease-targeted CRISPR trans reporter (DETECTR) described by Broughton et al. enables the rapid and straightforward diagnosis of SARS-CoV-2 RNA extracted from patient respiratory tract swab samples in less than 40 min. The diagnostic test combined CRISPR/Cas12a DETECTR system with RT-LAMP for purified RNA from nasopharyngeal or oropharyngeal swabs. Cas12a subsequently detects predetermined viral sequences followed by the cleavage of the reporter molecule, confirming the presence of the virus (Broughton et al., 2020).

The all-in-one dual CRISPR-Cas12a (AIOD-CRISPR) system designed by Ding and colleagues uses dual CRISPR RNAs (crRNAs) to efficiently detect the target genome sequence (both SARS-CoV-2 and HIV-1). The AIOD-CRISPR system combines all the components required for target nucleic acid amplification and CRISPR system-based detection into one reaction (Ding et al., 2020).

Yoshimi and colleagues developed an *in vitro* nucleic acid diagnostic tool based on Cas3, Cas3-operated nucleic acid detection N (CONAN). The CONAN tool was described as a fast, sensitive, and device-free diagnostic system for SARS-CoV-2 detection combined with isothermal amplification methods (Yoshimi et al., 2020).

The CRISPR/Cas12a-based system that allows reading with the naked eye (CRISPR/Cas12a-NER) described by Wang and colleagues was shown to detect at least ten copies of a viral gene in 40 min without the demand for specialized instruments. The designed system comprises Cas12 protein, SARS-COV-2-specific crRNAs, and a single-stranded DNA molecule as a reporter (labelled with a green fluorescent off–on molecule). When the coronavirus genome is present in the sample, it is detected by the designed diagnostic system - the reporter molecule is being cleaved by the Cas12 protein, triggering a green fluorescent light visible to the naked eye at 458 nm (Wang et al., 2020).

While a lot of research focus is targeted against the development of diagnostic systems based on SARS-CoV-2 nucleic acid detection rapidly and conveniently, only a few systems have addressed the issue of mutations and genomic rearrangements. Notably, RNA viruses frequently mutate to elude attacks from the host immune response. Multiple genomes of SARS-CoV-2 are continuously sequenced, and different mutations have been identified. For instance, mutations in the gRNA binding site can trigger mismatches that hinder the ability of CRISPR/Cas system’s ability to recognize the target area. The variant nucleotide guard procedure has been developed to address this issue and identify mutated and altered nucleic acid regions of SARS-CoV-2 (Ooi et al., 2020). The DETECTR system and different forms of Cas12a enzymes were initially analyzed, and among the tested molecules, enAsCas12a harboured the greatest tolerance in the CRISPR target area for single mismatches (Ooi et al., 2020).

#### Non-molecular POCT methods

POCT devices for the selective detection of biomolecules using metallic or magnetic nanoparticles are an important direction of research in the field of diagnostic optimization, the nanodiagnostic platforms having the ability to quickly detect, in real-time, the target biomarkers in minimal sample volumes. By comparison, conventional blood biomarker tests have lower sensitivity and require high concentrations of biomarkers. At the same time, while nanoparticle sensors present very high sensitivity and can detect the same biomarkers at concentrations thousands of times lower, thus enabling an early diagnosis (Wang et al., 2017).

POCT non-molecular technologies have two targets, respectively antibodies or antigens. One non-molecular POCTs is immunochromatography testing (ICT), which diagnoses several bacterial, viral, parasitic, and fungal infections. *Lateral flow immunochromatography* for *antibodies* detection has the most extensive use, despite the low sensitivity and the impossibility of concomitant analysis of multiple samples (multiplex).

The immunoassay POCT (lateral flow immunochromatography) applied to detect the influenza virus has a lower sensitivity and specificity and a narrower target spectrum than the molecular POCT. However, the diagnosis time is significantly shorter (15 minutes *versus* 30-60 minutes) (Egilmezer et al., 2018).

In the specific conditions of the pandemic with type A influenza (H1N1_2009_), detection of antibodies by lateral flow immunochromatography, although it did not have high sensitivity, was a valuable indicator for negative cases, thus, in the conditions of a high degree of contagiousness, decreasing the risk of contamination occurred in the analysis of suspected samples by NAAT. By extrapolation, this reasoning is also applicable to the SARS-CoV2 pandemic, with a very high degree of transmissibility.

Another category of the non-molecular POCTs are the immunofluorescence (IF) tests, providing the results in 15 minutes but with low sensitivity (~50%) for the influenza virus and even lower for the respiratory syncytial virus (RSV). The *Sophia Fluorescent Immunoassay Analyzer* works on the principle of lateral IF flow and consists of a kit for antigen detection and an optical sensor, providing the result in 10 minutes (Drancourt et al., 2016).

Other non-molecular POCTs are based on the detection of antibody using different *ELISA* (enzyme-linked immunosorbent assay) protocols, silver-coated microspheres, or the protein array technology. In the case of the two-photon excitation assay technique and dry-chemistry reagents, the virus is sandwiched with polymer microspheres and fluorescently labelled antibodies. Immunocomplexes are formed on the surface of the microspheres, proportional to the concentration of the analyte in the sample. The fluorescent signal emitted by the microspheres is measured using two-photon-excited fluorescence detection (Koskinen et al., 2007).

In surface plasmon resonance (SPR), biomolecules bind to a metal surface and absorb a part of the incident light flux, leading to decreased reflection. Qiu and colleagues described a dual-functional plasmonic biosensor combining the plasmonic photothermal (PPT) effect and localized LSPR as a promising solution for diagnosing SARS-CoV-2 infection. The two-dimensional gold nanoislands functionalized with complementary DNA receptors performed a sensitive detection of the selected sequences from SARS-CoV-2 through nucleic acid hybridization (Qiu et al., 2020).

### Biosensors-Based POCT

**Figure 1:**
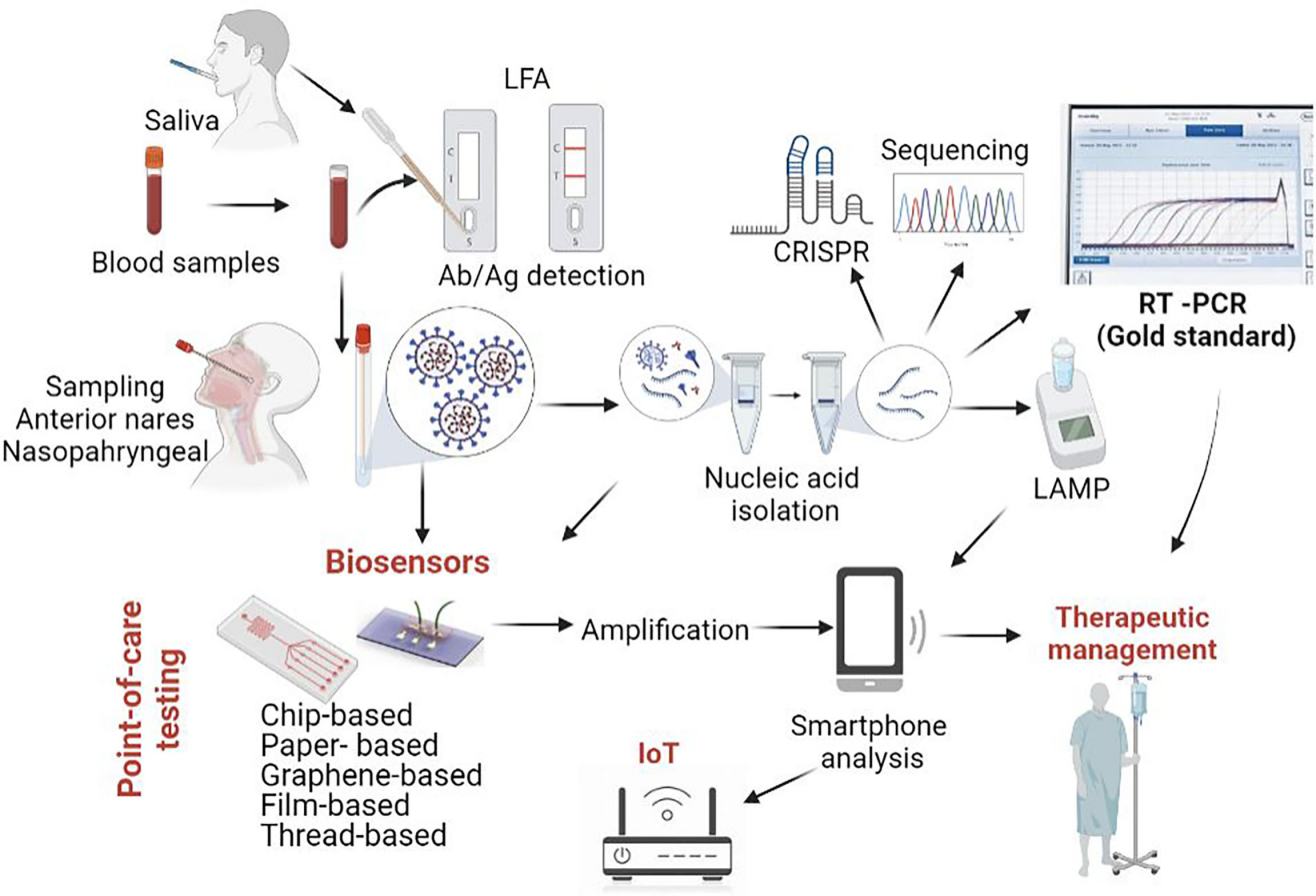
SARS-CoV-2 diagnostic tools. While Real Time PCR from nasopharyngeal swabs represents the gold standard from COVID-19 diagnostic, other approaches are currently being employed or developed. Among these, research is focused on various types of biosensors (chip based, paper based, graphene-based biosensors), some of them coupled with smartphone analysis. Original figure, created using biorender.com.

Recently, biosensors have gained significant interest due to their ability to provide rapid, portable, sensitive, miniaturised, and inexpensive alternative diagnostic platforms. Typically, a biosensor is made of three parts: a “bioreceptor” unit (DNA, enzyme, antibody) combined with ion conductive materials able to recognize the analyte, a physio-chemical signal transducer (optical, electrochemical or piezoelectric) and a reader device (Afroj et al., 2021). Biosensors can be designed to detect viral antigens, antibodies produced against a virus or the virus genome. Viral antigens are easier to detect since they are displayed on the outer surface of the virus and can strongly bind to the biosensor receptors or antibodies. Notably, the efficient liquid mixing in biosensors favours the interaction between assay reagents and the target biomarkers, shortening the assay duration and providing a fast readout (Rasmi et al., 2021). Until recently, most biosensors developed focused on detecting Influenza due to the many subtypes of this virus and increased mortality and morbidity (Dziąbowska et al., 2018). However, the emergence of the SARS-CoV-2 pandemic has significantly moved the focus on this new virus. Indeed, many biosensors have been designed in the last two years for the diagnostics of COVID-19. For instance, chip-, paper-, and graphene-based biosensors have been developed as complementary diagnostic modalities. Furthermore, connecting the devices to smartphone could become an easy and rapid analysis method for remote diagnosis, data collection, and disease monitoring. An overview of the entire process, from sample collection and detection to distant communication and disease management with possible POCT application is described in **Figure 1**.

The development of diagnostic systems to customize the assessment of the pathophysiological condition is the next stage in the development of POC systems. POC biosensors are made of poly-dimethyl-siloxane (PDMS), paper and other flexible materials such as textiles, film and carbon nanosheets (Choi, 2020). The analytical performance of POC systems can be evaluated by electrochemical biosensors, which are components of POC devices. The biosensor estimates the quantitative level of biomarkers based on a specific chemical reaction and generates a quantifiable signal: the signal strength is proportional to the concentration of the analyte in the sample. Thus, the condition can be diagnosed based on the signal of body fluid markers) (Noah and Ndangili, 2019).

The electronic circuit, transducer element and the recognition section are the three main components of a biosensor. There are different types of biosensors: electrochemical, optical, electrical, thermometric, and piezoelectric. In the case of electrochemical biosensors, there have been multiple transistors used, including conductometric, potentiometric, amperometric, voltametric and impedimetric. The types of electrodes vary as well, and they can be solid (gold, platinum, diamond, or carbon) or composite electrodes. There are three types of electrodes for conducting these techniques: reference, working and auxiliary. These electrodes can use various biological elements that they can recognize, such as tissue, antibodies, nucleic acids, enzymes, organelles, cell receptors. Electrochemical biosensors are investigated for their use in POC technology, with conductometric biosensors being seen as the best candidate for this (Anik, 2017; Tepeli and Ülkü, 2018; Qazi and Raza, 2020).

Electrochemical biosensors have high sensitivity and accuracy, low detection limits and high real-time analysis potential. These biosensors offer many electrode materials and target detection molecules with multiple modification methods. Importantly, these sensors can successfully be exploited to detect different viruses by changing the probe immobilized on the electrode surface. The gold-coated array of carbon electrodes was the method of identifying the S protein of MERS-CoV in 20 min. In the case of SARS-CoV infections, the specific antibodies competitively bind the viral particles present in the sample, or the viral antigen immobilized on the electrode bind the serum antibodies. A peak current through the chip measures the antibody or antigen bound on the electrode (Nelson et al., 2020). A chip-based electrochemical biosensor made of ion-sensitive field-effect transistors (ISFETs) using complementary metal-oxide-semiconductor (CMOS) technology was developed for SARS-CoV-2 detection. The platform was reported to harbour a detection limit of 10 copies of SARS-CoV-2 RNA per reaction with 90.55% sensitivity and 100% specificity (Rodriguez-Manzano et al., 2020). The sample preparation (nucleic acid isolation and LAMP amplification) was done off-chip, and the amplicons were added into the developed biosensor for nucleic acid detection. The voltage change triggered by the pH variations was recorded and analysed with the help of a custom smartphone Android OS application. Even though this biosensor has clear advantages related to costs and portability and provides quantitative data, it could be further improved by eliminating the sample preparation step. An electrochemical biosensor made of a three-electrode system including platinum reference electrodes, a counter electrode and a titanium substrate functionalized with gold nanoparticles was developed for COVID-19 diagnostic. The sensor contained a single-stranded probe with a thiol end immobilised on the gold surface, which was complementary to target viral nucleotides. The main disadvantage of this assay was again related to the need for sample preparation -isolation of target DNA/RNA- (Tripathy et al., 2020). From this perspective, simplified techniques were approached for ready-to-use small volumes of biological samples. Fabiani and colleagues developed an electrochemical sensor for saliva testing using screen-printed electrodes and magnetic beads with selectivity for SARS-CoV-2 spike protein (19 ng/mL) or nucleocapsid protein -8 ng/mL- (Fabiani et al., 2021), while Ahmadivand and colleagues described a toroidal plasmonic immunosensor to detect SARS-CoV-2 spike protein at femtomolar level with POCT potential (Ahmadivand et al., 2021). Dalal and colleagues employed immobilised HA gene-specific oligoprobe and developed a genosensor to detect sensitive POC H1N1 (swine flu) in human nasal swabs (Dalal et al., 2020). The complementary ssDNA probe immobilized on a cysteine-functionalised screen-printed gold electrode generated an electrochemical differential pulse voltammetry signal upon hybridisation to the target viral genome in the presence of a redox indicator -methylene blue- (Dalal et al., 2020).

Another type of technology that offers a variety of advantages is *microfluidic sensors*. Paper-based microfluidic POC sensors are based on colorimetric and electrochemical assays, and they present the great advantage of having a widespread application, from the environment to food and human health. Since biosensors present various advantages, such as their high sensitivity, specificity, and user-friendliness, the biosensor-based devices can be integrated with different types of material, including paper, silicon, and magnetic beads. Nanomaterials are combined to give these electrodes more power (Li et al., 2017; Zhang et al., 2017). *Nanotechnology* is the field that uses the atomic and molecular levels to create systems with physical, chemical and biological applications, with nanoparticles (nanomaterials) achieving dimensions of 1-100 nm. *Nanodiagnostics* is defined as the use of nanotechnology in diagnostic applications, including biosensors (e.g., magnetic, silver and gold metallic nanoparticles) for the diagnosis of infectious diseases. Nanostructures have a very high surface/volume ratio and are very suitable for binding many target molecules to immobilised probes, which increases the sensitivity of the detection results (Brazaca et al., 2017; Younis et al., 2020). Biosensors based on metallic nanoparticles are often used due to their unique optical properties. Gold is generally used for biosensor development due to its biocompatibility and surface chemistry. Colorimetric detection based on gold nanoparticles (AuNPs) takes advantage of the colour change from red to blue due to localized surface plasmon resonance (LSPR) coupling among the nanoparticles (Li et al., 2015). However, researchers claim that nanomaterials can be problematic due to their inconsistent signal amplification and metallic impurities (Kuila et al., 2011).

Fluorescence chip-based biosensors are also promising diagnostic tools. For instance, Sun and colleagues developed a fluorescence-based biosensor that allowed the detection of Equine herpesvirus 1 (EHV1) from horse nasal swabs in 30 min with a detection limit of 18 copies per reaction. In this paper, EHV1 served as a model system for diagnosing SARS-CoV-2: the amplification primers were injected into the microfluidic channels and dried, the sample was introduced to the channel, followed by LAMP amplification at 65°C. After LAMP, fluorescence images were captured by a smartphone. The display was based on the emission of the DNA-intercalating dye EvaGreen to obtain an average pixel intensity value for detecting target nucleic acids (Sun et al., 2020).

Ganguli and colleagues elaborated a fluorescence chip-based biosensor based on LAMP for real-time detection of SARS-Cov-2 (with a detection limit of 5000 RNA copies/μL in nasal samples). The chip was made from 3D-printed microfluidic polymer cartridges consisting of a heater and optics *via* syringes and used EvaGreen, a double-stranded DNA-intercalating dye. The fluorescence signal generated by the amplicons was recorded *via* a smartphone, followed by analysis using ImageJ software (Ganguli et al., 2020).

Liu and colleagues developed a biosensor based on a fluorescent immunoassay to detect IgM, IgG, and SARS-CoV-2 antigen simultaneously. Each analyte is selectively detected on an individual microfluidic chip while they are simultaneously read in a portable device combining liquid handling and signal readout for POC diagnostics (Liu et al., 2020). Importantly, simultaneous detection of viral antigen and corresponding antibodies gives a specific and accurate diagnosis of SARS-CoV-2, overcoming barriers such as the transient expression of IgM in the blood (Lee et al., 2020) and the cross-reaction of antibodies targeting various coronavirus strains (Yuan et al., 2020). Chen and colleagues designed a lateral flow immunoassay to test for the presence of anti-SARS-CoV-2 IgG antibodies in serum samples. The test comprised lanthanide-doped polystyrene nanoparticles (L-NPs) acting as fluorescence reporters functionalized with either mouse anti-human IgG (M-HIgG) or rabbit IgG (rIgG) antibodies. As human serum was loaded onto the flow assay, human anti-SARS-CoV-2 IgG antibodies conjugated with M-HIgG@L-NPs and attached to the test line material leading to a change in the fluorescent signal at the test line (Chen et al., 2020).

Paper-based biosensors such as lateral flow test strips are versatile, cost-effective, and widely used in the clinical setup. Indeed, paper-based sensors have been used for the detection of cancer biomarkers as well as for the diagnostic of viral infections such as Ebola (Brangel et al., 2018), influenza A H1N1 (Lei et al., 2015) and recently, SARS-CoV-2 (Li et al., 2019). Paper-based biosensors can be classified into dipstick assay, lateral flow assay (LFA) and microfluidic paper-based analytical devices (μPADs). Generally, LFA is frequently used for pathogen detection due to their user-friendliness, low cost, and relatively high sensitivity. The paper substrates routinely used are cellulose (dipstick, μPADs) and nitrocellulose (LFA) membranes (Hu et al., 2014).

Wu and colleagues (Wu et al., 2017) developed an automated and portable paper-based microfluidic system for Influenza diagnosis, which consisted of a storage module with reagent chambers and a reaction module with the absorbent pad and nitrocellulose membrane functionalized with specific monoclonal antibodies. A smartphone was used to capture the image from the membrane and process the image with a Java algorithm.

Photonic biosensors have a high signal-to-noise ratio, and they can convert the molecular binding events to optical signals, easing the integration into mobile phones. For example, Su and colleagues developed a portable module that can detect clinically relevant levels of a secretory leukocyte protease inhibitor, an established biomarker for a lung infection in cystic fibrosis patients. Moreover, the results can be interpreted instantly and transmitted to the medical personnel (Sun et al., 2016).

Graphene-based biosensors have a higher electron transfer rate and a larger electrochemical surface area exhibiting advantages such as low cost and high production rate. A graphene-based FET biosensor was developed for SARS-CoV-2 diagnostics, using spike protein antibodies as the detection probe. Sensor performance was tested on a cultured virus, antigen protein and clinical nasopharyngeal samples and showed detection of SARS-CoV-2 spike protein at concentrations of 100 fg/mL in clinical transport medium and 1 fg/mL in phosphate-buffered saline (Seo et al., 2020). Mojsoska and colleagues reported the first optimization steps of a label-free electrochemical immunosensor using a graphene-modified working electrode to detect the SARS-CoV-2 spike protein. The biosensor detected subunit 1 (S1) of recombinant spike S protein at 260 nM (20 µg/mL) of subunit 1 of recombinant spike protein and SARS-CoV-2 (5.5 × 10^5^ PFU/mL) (Mojsoska et al., 2021).

### Integrated Systems With Potential for POC Use

Future devices will need to be ultra-sensitive and accessible for POCT detection of respiratory viruses (Noah and Ndangili, 2019). In this regard, the use of multiplex, integrated LOC systems will allow the simultaneous identification of several pathogens in a sample, although coinfection is not common in the case of viral RTI. Integrated systems should operate on a ‘sample in and answer out’ principle. During the early stages of infection, the biomarkers of viral infection have very low concentrations, and in conditions of the low sensitivity of POCT, the result will be negative. Enriching the biomarkers or the virus in the sample before detection is necessary (Yeh et al., 2014). Examples of integrated systems with potential POC use include the Alere BinaxNOW^®^ (formerly Alere i) Influenza A&B platform (Abbott, United States; uses a fluorescent molecular signal) and the FILMARRAY Respiratory Panel (bioMérieux, France). The FILMARRAY Respiratory Panel employs nested real-time PCR to detect twenty respiratory pathogens. Seventeen viruses (AH1, AH1 2009, AH3, influenza B, adenovirus, the four common coronaviruses that produce the common cold, human metapneumovirus, human Rhinovirus/Enterovirus, parainfluenza 1, 2, 3, 4 and RSV) and three bacteria (*Bordetella pertussis*, *Chlamydophila pneumoniae*, and *Mycoplasma pneumoniae*) are detected. The specimens are from buccopharyngeal swabs, nasopharyngeal aspirates, and lower respiratory tract samples. The FILMARRAY Respiratory Panel platform has the precision of PCR diagnosis, it can be used as a POCT, it does not require laboratory specialists, and the results are available in one hour. MariPOC system (produced in Turku-Finland) is a multianalyte detection system that can simultaneously detect nine respiratory viruses (influenza A and B, RSV, parainfluenza 1, 2, 3, metapneumovirus, HboV - a parvovirus, adenovirus) and *Streptococcus pneumoniae*. (Bruning et al., 2017) Compared to RT-PCR, it presents moderate sensitivity for influenza and RSV and low sensitivity for adenovirus (Ivaska et al., 2013).

The GeneXpert systems automate and integrate sample preparation, nucleic acid extraction and amplification, and detection of the target sequences using real-time PCR and RT-PCR assays (Cepheid, a Danaher company, United States). The system runs on disposable (Xpert) cartridges that contain the RT-PCR reagents and allow the RT-PCR process. The GeneXpert panel Xpert Xpress SARS-CoV-2/Flu/RSV test is a rapid, multiplexed real-time RT-PCR test designed for the simultaneous qualitative detection and differentiation of influenza A, influenza B, SARS-CoV-2, and respiratory syncytial virus (RSV) viral RNA in a nasopharyngeal swab, nasal swab or nasal wash/aspirate (Mostafa et al., 2021).

### POCT Performance and Unmet Needs

Only a rapid, sensitive, and specific identification of pathogens will allow effective antiviral therapy and enable infection control measures. Even though POCT are simple to operate, correct sample collection, conditioning and/or preparation is essential to improve test performance. Moreover, multiple factors hindered the performance of POCT, such as the quality and handling of the specimen and the respiratory specimen type. Respiratory viruses are more likely to be detected when patient samples are collected soon after symptom onset because viral loads are generally higher early in the illness, with viral shedding peaking in the first 2-3 days of illness in adults (Foo and Dwyer, 2009). However, in children, viral shedding can take longer (Hazelton et al., 2015).

Because POCT can be performed outside the laboratory setting, they are not subject to quality assurance requirements. These rapid tests are approved by governing bodies (i.e., FDA), but their manufacturers are not obliged to monitor and improve their diagnostic tests. (Gill and Shepard, 2010) Hence, the performance of POCT may diminish once antigenic and genetic variants emerge. Continuous monitoring of POCT is of paramount importance to provide clinically relevant results.

While POC biosensors are widely available, there are still some unmet needs. For instance, commercial diagnostic kits involving IgG/IgM test strips need improved sensitivity and specificity. In addition, the multiplexing capability is essential for improved diagnosis and subsequent therapeutic management. For instance, the combination of both IgG/IgM and nucleic acid tests would detect both early and late stages of infection (such as COVID-19), thus yielding more accurate and reliable results.

Currently available POCT can be improved by incorporating antiviral susceptibility testing and identifying coinfections. For example, Hwang and colleagues described a lateral flow assay capable of detecting Tamiflu-resistant influenza virus using oseltamivir hexylthiol AuNPs with binding selectivity to Tamiflu-resistant virus. The test comprised detection and a control line marked with anti-influenza A virus nucleoprotein antibody and Tamiflu resistant neuraminidase protein (Hwang et al., 2018). This type of approach could potentially be developed for SARS-CoV-2 infection as well.

Another point for POCT improvement would be using alternative samples for diagnostic. For instance, respiratory droplets and aerosols are the main transmission vessel for respiratory infectious diseases but they are rarely used for diagnostic means (Tromberg et al., 2020). An interesting recent study by Nguyen and colleagues (Nguyen et al., 2020) described the use of wearable materials with embedded synthetic sensors for biomolecule detection, including SARS-CoV-2 (Nguyen et al., 2020). The research group developed a face mask sensor because viral particles accumulate inside masks because of respiration, sneezing, talking and coughing. The biosensor was made of four modular components, including a large surface collection sample pad, a reservoir for hydration, a lateral flow assay strip and a wax-patterned µPAD. All collected fluid and viral particles from the sample were shown to migrate from the sample collection pad to the µPAD, carrying an arrangement of freeze-dried lysis and detection components (Nguyen et al., 2020).

It is essential to highlight that POCT provides test results with a faster turnaround time in most instances compared to traditional approaches informing the clinical personnel before the patient reaches the hospital. Although detection of a virus does not exclude the possibility of a bacterial infection or a benefit from antibiotic treatment, a positive POCT result might also permit the premature discontinuation of precautionary antibiotics in patients with exacerbation of airways disease. If negative, a POCT result can shorten unnecessary quarantine measures and the use of antiviral treatments. Even though they are important tools in diagnosis, rapid tests do not necessarily guarantee improved clinical outcomes for all patients (Crozier et al., 2021).

## Internet-of-Things in Detection of Respiratory Viral Infections’ Detection

IoT is an application-specific, low power, effective, and easy to use tool to provide solutions to any real-time problems. Since IoT means are approached from various points of view, billions of connected things are already in use in 2015, and that number will reach 25 billion in just a few short years (Middleton et al., 2013). In the IoT world, sensors provide inputs from the physical world, which are transferred over a network, and actuators allow devices/things to act or react according to the received inputs. Gil and colleagues reviewed the surveys related to IoT, their general purpose and provided a well-integrated perspective for IoT, including a state-of-the-art of IoT to integrate IoT and social networks in the emerging Social Internet-of-Things (SIoT) term (Gil et al., 2016). Meanwhile, Internet-of-Medical-Things (IoMT) (Gatouillat et al., 2018) generates and aggregates abundant data in various ways. For instance, medical devices track the physiological parameters of patients (Bharadwaj et al., 2021) from home and the remote healthcare providers receive information about the mobility, slipping dynamics, heart rate, allergic reactions, blood pressure, blood glucose, body temperature, and oxygen saturation of the patients (Patel and Wang, 2010; Fortino et al., 2014; Jayanth et al., 2017), and decide on an accurate diagnosis (Panesar, 2019), build therapeutic plans, improve the security of patients, simplify caregiving, and continuously monitor critically ill patients (Gómez et al., 2016). Online consultations with doctors *via* Telehealth (Craig and Petterson, 2005) and imaging investigations (Huang et al., 2020) are more examples of IoT in healthcare, which contribute significantly to the quality of medical care and wellbeing of patients whose health status prevents them from going to hospitals. Additional modalities of remote healthcare *via* IoT medical devices facilitate personal emergency response systems (PERS). The solutions can make automatic calls for help in case of sudden changes in the medical status of the patients that make them appear at risk of being unable to call the ambulance on their own. Furthermore, in the case of infectious diseases such as COVID-19 remote assistance and testing can significantly reduce hospital occupancy rates during the pandemic (Tuli et al., 2020). Currently, several IoT devices and applications are being employed in COVID-19 management, including smartphone applications, wearables, drones, IoT buttons and robots. Their characteristics are summarised in **Supplementary Table 3**.

A real-time response should follow a rapid diagnostic of viral RTI. Current advances in information technology exploit IoT related equipment and offer a large capacity for pandemic monitoring. For instance, smartphones are equipped with “lab-in-a-phone” IoT devices and an instant global positioning system (GPS) for rapid contact tracing (Xu et al., 2018; MD-Bio, 2021). There is an excellent opportunity to integrate IoT-POC connected devices by using cloud-based connectivity, and thus the results of the immediate analysis will be delivered to control centres for disease monitoring. Newly developed swarm technologies will develop real-time maps of the spread of infectious at the global level.

**Figure 2:**
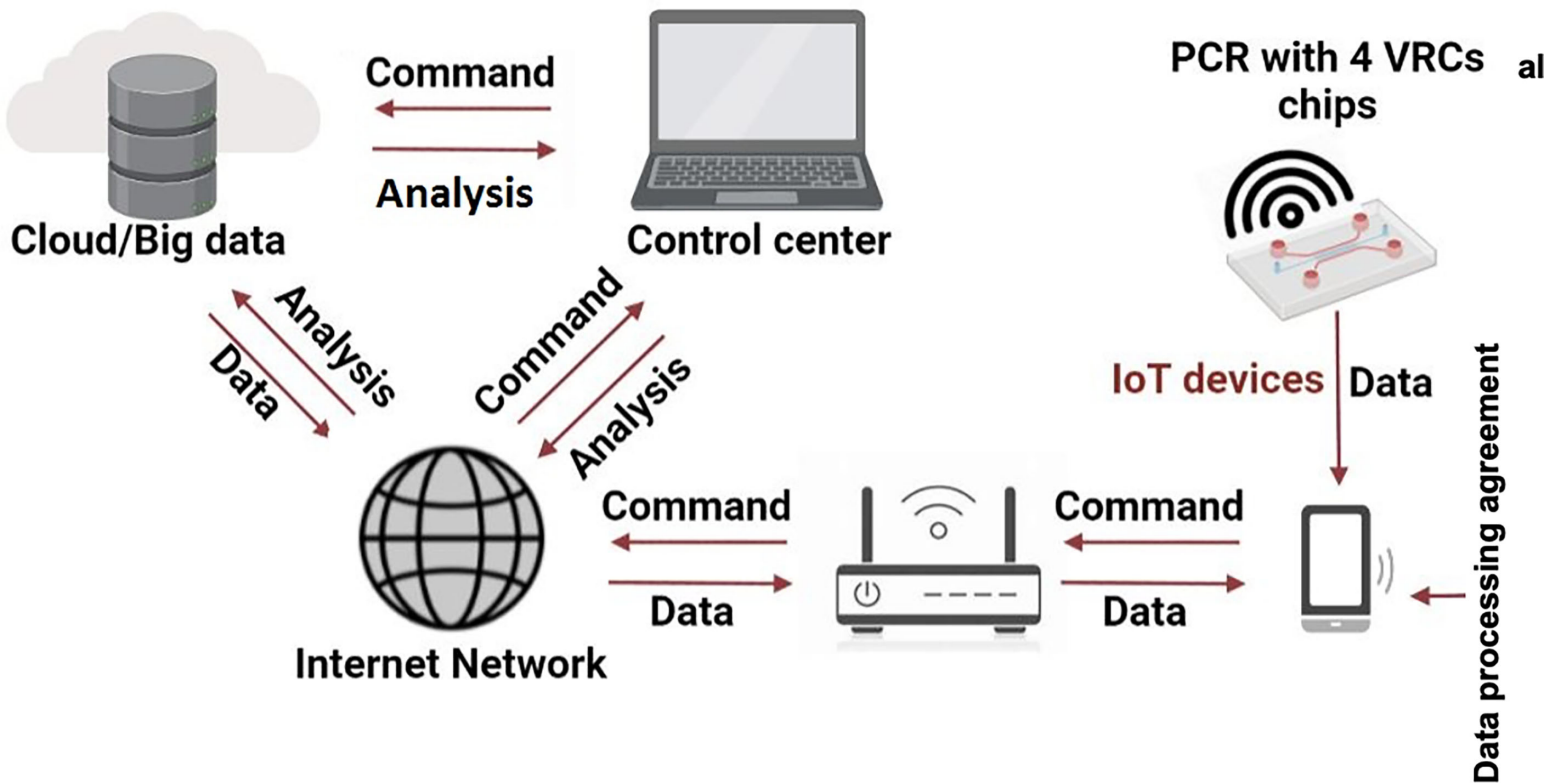
Working principle of an IoT PCR for RTI detection and spread monitoring. Adapted with permission from (Zhu et al., 2020).

The first step in this direction was a hand-held IoT PCR used for dengue fever detection and its spread monitoring reported by Zhu and colleagues (Zhu et al., 2020). Such an IoT system is illustrated in **Figure 2** -adapted and improved from (Zhu et al., 2020). The sample tested for dengue fever can be processed anywhere, and the results and GPS location are submitted wireless using the patient’s handphone to a control centre. A network can collect all results as cloud data to map a disease outbreak, show the respective area and continuously monitor. Surely, the solution can raise the question about the cybersecurity.

The IoT based biosensor ‘RapidPlex’ was grafted for ultra-rapid assessment of COVID-19 biomarkers (Torrente-Rodríguez et al., 2020). RapidPlex is a multiplex electrochemical platform that quantifies four COVID-19 markers: nucleocapsid protein, IgM, IgG antibodies and inflammatory C-reactive protein.

Recently, Mukhtar and colleagues described a device for measuring patients’ critical status of the effects of SARS-CoV-2 infection or its symptoms using cough, temperature, heartbeat, and oxygen concentration. The device comprises wearable medical sensors integrated using the Arduino hardware interfacing, a smartphone application and an IoT framework (Mukhtar et al., 2021).

Kumar and colleagues presented a list of IoT sensors and their application areas and proposed an IoT architecture to avoid the spreading of COVID-19 (Kumar et al., 2020). Sensors in crowded places such as airports, malls, transport, public toilets, hospitals, and offices communicate data through a gateway device to cloud gateway and the big data used by machine learning (ML) will create models of the system based on requirements and received data. The configuration includes one thermo-sensor, one NodeMCU or Arduino board with sensors, the Internet, and possibly one mobile application at a smaller scale. Several open sources can be used to implement the protocol. Furthermore, Peeri and colleagues presented the role of big data in understanding the epidemiology and clinical factors that characterise the MERS, SARS and COVID-19. (Peeri et al., 2020) In terms of SARS-CoV-2 infection diagnostics, Lopez-Rincon and colleagues (Lopez-Rincon et al., 2020) proposed a deep convoluted neural network (CNN) to create features from genome sequencing automatically. The accuracy of identification and classification into different coronaviruses were 98% and 98.75%, respectively, despite the limited data set and limited genome sequences being considered. A model that demonstrated the superiority of a Support Vector Machine (SVM) classifier in identifying influenza-like illnesses -i.e., acute respiratory infections- (Ginantra et al., 2020) used neural networks, level 2 models with better results than a single level model. The design stemmed from the rapid antigenic shift as the source of virus variants makes the understanding of effects of specific mutations on pathogenicity difficult to understand. The results for a confusion matrix showed 97.4% sensitivity, 90% specificity, and for the K-Nearest Neighbours (KNN) classifier, 94.7% accuracy. Pineda and colleagues studied seven ML classifiers for influenza detection, compared their diagnostic capabilities against an expert-built influenza Bayesian classifier, and evaluated different ways of handling missing clinical information from the free-text reports in four hospitals emergency departments (Pineda et al., 2015). The study, despite its limitations, showed that ML classifiers had a better performance than expert constructed classifiers are given a particular natural language processing (NLP) extraction system and that analysing information from the electronic health records using machine learning classifiers can achieve significant accuracies in the presence of abundant clinical reports. Since large data is crucial to the early identification of acute upper RTI during early stages, it minimizes adverse effects on infants and prevents mortality. Therefore, studies addressed disease prediction of cases. For instance, Yin and colleagues established a stacking model to predict on antigenic variants of the H1N1 influenza virus (Yin et al., 2018). The study first classified past cases as pandemic-based and epidemic-based and concluded that ML classifiers showed superior performance to expert Bayesian classifiers for the given use case. Sato and colleagues built an epidemic spread model using IoT technologies to monitor human mobility and contact data (Sato et al., 2016). They introduced the agent-based infections diffusion simulation using real human mobility data as a metapopulation network to control the spread of outbreaks. A study by Chen and colleagues used mobile phone data to build a model to track dynamic changes in a network as an epidemic spread network (Chen et al., 2016). The control of epidemics was also addressed by Miller and colleagues who proposed methods to generate accurate forecast data for influenza outbreaks using smart thermometers connected to a mobile application (Miller et al., 2018). The data aggregated and stored onto a cloud-based platform, analysed in conjunction with the Centre for Disease Communication and users’ location, developed a model for forecasting ILI up to three weeks in advance and helped track fever duration to identify biphasic patterns. Referring to influenza outbreaks, Tapak and colleagues investigated different ML methods for building models on illness frequencies (Tapak et al., 2019). However, not including factors of climatic parameters, weather conditions, and sentinel data from ILI limited the and asked for careful evaluation before implementing the algorithm for predicting the outbreaks of the disease. Since the limitations of these studies needed to be addressed, significant effort concentrated on solutions such as remote labs to complement the control engineering laboratories and include industrial revolution relevant methods. For instance, an open-source feature with LINX and Arduino board was incorporated to assist in the data acquisition with cost-effective platforms that interact with the actual labs *via* mobile devices (Ferrari et al., 2017; Andreini et al., 2018; França et al., 2021; Hairuddin et al., 2021). Furthermore, IoT enables further development of remote medicine for the on-body monitoring *via* either consumer wearables (track the health conditions of patients and healthy people) or clinical devices -track and transmit some highly specialised health metrics directly to healthcare organisations and doctors- (Atzori et al., 2010). Notably, these devices can be customised for other viral agents. Hence they harbour tremendous potential in the management of infectious diseases. Despite their considerable advantages, IoT devices raise concerns regarding the privacy issues occurring when patients are asked to share their information. Clearly, defining secure channels for communications or different encryption techniques before sharing private information is essential for the successful implementation of IoT devices. Therefore, optimising nano-enabled viral biosensing, IoT, and artificial intelligence (AI) could open avenues for various integrated low cost highly performant detection technology for error-free, smart controlling at a personalised level. In conclusion, IoT technology coupled with POCT platforms would be instrumental in offering diagnostic platforms and therapeutic approaches for future global health challenges (Konwarh and Cho, 2021, Swayamsiddha et al., 2021).

## Next Step in the Detection of Respiratory Viral Infections - AI

Internet-connected POC testing devices, coupled with predictive models and artificial intelligence (AI), would be a great asset in disease evolution monitoring and provide a valuable tool to stop their spreading (Sim and Cho, 2021). AI programs developed to diagnose a disease would be trained on data-like symptoms, lab results, and scans and images of confirmed and susceptible cases. AI-based detection techniques can be successfully employed for diagnosis. For instance, end-to-end portable systems can record data from symptomatic patients (*i.e.* coughs) and further translate them into health data for diagnosis. Subsequently, with ML symptoms can be linked to different respiratory illnesses, including COVID-19.

## Conclusions

Recently, significant improvements in respiratory virus diagnostics, from novel specimen collection instruments to highly sensitive and multiplexed nucleic acid amplification tests, demonstrated the potential of the expanding list of antigen and molecular-based tests for comprehensive laboratory testing of respiratory viruses without even virus isolation. However, cell culture remains the reference method with excellent sensitivity and specificity (detects a broad spectrum of viruses as little as 1 infectious unit). Compared with cell-culture-based viral load quantification, immunofluorescent staining provides results within several hours, but the sensitivity and availability of antisera can be limiting factors. Furthermore, PCR assays with superior sensitivity to culture or immunofluorescent staining have been transformed into an essential viral diagnostic tool. Also, the main challenge NAAT technology faces is to achieve inexpensive, high-throughput, and automatic NA detection from raw samples (e.g., whole blood). Therefore, most diagnostic virology laboratories use a combination of viral culture, immunofluorescent staining, and PCR assays to detect respiratory viruses. The continuous advances of technology to improve turnaround time (combined shell vial cultures and immunostaining) and sensitivity (nucleic acid amplification technologies such as PCR and LAMP) has paved the future of additional commercial test kits for molecular detection of respiratory viruses, including multiplexed assays.

Moreover, further research is required to elucidate the clinical significance of persistent positive PCR results in a patient and viral coinfection. Ultimately, the utilisation of molecular testing, particularly highly multiplexed tests in routine patient management, will depend on the cost/benefit ratio. The solution might be the fully integrated microfluidics devices easily manufactured and used (Li et al., 2020, Nelson et al., 2020). Therefore, cost-effective LOC platforms (microfluidics- and biosensors-based) as future technology developments in respiratory virus diagnostics should accurately and rapidly detect a spectrum of clinically significant viruses to influence the patient and epidemiologic management at higher standards for systematic implementation of standardised infection control measures not achievable with laboratory-developed tests. The current COVID-19 pandemic highlights again the stringent need for tools and technology for fast, accurate, non-molecular and molecular POCT cum IoT devices for efficient diagnosis. Other studies revealed the applicability of digital PCR (Chen et al., 2021), surface-enhanced Raman scattering -SERS- (Chen et al., 2021), plasmonic- (Li et al., 2019) and nanophotonic label free-based biosensors (Soler et al., 2020) in nucleic acid rapid testing and respiratory viral disease diagnosis in the future.

Additionally, their large-scale implementation will require laboratory validation and quality assurance protocols as the performance will vary with each generation of assays and clinical environment (Bouzid et al., 2021). It is also crucial that specialists understand the characteristics and limitations of commercial kits provided to keep the costs affordable. Since novel viral outbreaks are foreseeable, it is imperative to have portable, fast, and accurate virus sensing technology for timely diagnosis. Virus identification can be used to (1) determine treatment strategies (antiviral medications), (2) predict disease course and expected outcome, (3) predict the potential for virus spread, (4) allow identification and vaccination of susceptible individuals, and (5) trace the movement of a virus through a community or worldwide. The efforts invested into this rapidly developing domain should continue and materialise.

## Author Contributions

GP, FI, MP, MS, GM, AC, OI, LB, and MT wrote the manuscript. MC and CI revised and edited the manuscript. All authors contributed to the article and approved the submitted version.

## Funding

This research was funded by the grant PN-III-P2-2.1-SOL-2020-0090-Contract 13Sol/15.06.2020, project “Advanced techniques for early SARS-CoV2 detection” and PN-III-P4-ID-PCE-2020-1886 Contract PCE 180/17/02/2021 awarded to CI and by CNFIS-FDI-2021-0405, awarded to MC.

## Conflict of Interest

The authors declare that the research was conducted in the absence of any commercial or financial relationships that could be construed as a potential conflict of interest.

## Publisher’s Note

All claims expressed in this article are solely those of the authors and do not necessarily represent those of their affiliated organizations, or those of the publisher, the editors and the reviewers. Any product that may be evaluated in this article, or claim that may be made by its manufacturer, is not guaranteed or endorsed by the publisher.
