# Supplementary material for: Advances in the Rapid Diagnostic of Viral Respiratory Tract Infections
Source: Front Cell Infect Microbiol. 2022 Feb 10;12:807253. doi: 10.3389/fcimb.2022.807253 (PMC8895598; doi:10.3389/fcimb.2022.807253)
Supplement: Supplementary file 1 [file Table_1.pdf]

TABLE 1: Main diagnostic methods in virology

Methods to diagnose viral infections	Method	Principle and Application	Advantages	Limitations	Protocols
Conventional					
Infectivity assays (cell culture-based)	Virus plaque test: (Leland and Ginocchio 2007, Mendoza et al., 2020)	Quantitative detection (virus multiplication, and isolation) of viruses forming plaques on the monolayer of cells in cell culture plates	- It is the gold standard for the virus isolation.<br>- With TEM is used during large scale outbreaks with new viruses (Johnson et al,. 1977, Drosten et al., 2003, Harcourt et al., 2020)	- It is limited to only a subset of animal viruses that can lead to cell lysis.<br>- Has false negatives from difficult-to-culture viruses (coronaviruses, MPV, many serotypes of human rhinovirus HRV) or unknown viruses when cell lines are chosen incorrectly. (Henrickson 2004, Van Den Hoogen, Osterhaus et al. 2004)<br>- It is time-consuming, laboratory-based, expensive	- For virus confirmation:<br>Cell culture + TEM (Goldsmith and Miller 2009);<br>Cell culture + Hemagglutination assay.<br>-For virus identification:<br>Cell culture + immunofluorescence staining (Amarilla et al., 2021)
	Shell vial (rapid cell culture)	A platform including the specimen inoculation on the cell monolayer grown on a coverslip, low-speed centrifugation, and detection of the virus by immunofluorescence	rapid technique to isolate Influenza, and other respiratory viruses(Lu et al., 2021).	Has insufficient sensitivity	For virus detection: Rapid cell culture + immunofluorescence
	Viral flow cytometry	Direct or indirect virus quantification using specific cell constituent dyes, antibodies to identify virus proteins and subtypes (Schulze-Horsel et al., 2008, Soni et al., 2020)	Has relatively short turnaround time.<br>It is easy to interpret the results.<br>It can be automated.	-It has insufficient sensitivity and specificity.<br>-It requires sophisticated equipment and specific training to use it.<br>-The primary antibody needs to be specific	- Coupled with fluorescence microscopy, and confocal microscopes, as imaging flow cytometers, it is typically a more practical and faster method of visualizing viral infections. (McClelland et al., 2021)

TABLE 1: Main diagnostic methods in virology

	Transmission electron microscopy (TEM)	A beam of electrons transmitted through a specimen for intracellular imaging	-Directly images the viral particles.<br>-Elucidates structures and conformations of complex and dynamic proteins without crystallization in their native, fully functional states	The sample preparation for TEM is laborious	It follows cell culture for virus identification (McClelland et al., 2021)
Serological methods (immunoassays)<br>Rapid detection of known viruses with known viral proteins: serotyping (detection of viral antigens in serum) and serodiagnosis (detection and quantification of specific antibodies in bodily fluid)	Radioimmunoassay (RIA) (Kim et al., 2021)	Radioisotopes-labelled immune reaction to viral specific immunoglobulins or antigen levels	-Detects either antigens or antibodies with high sensitivity.<br>-Differentiates between exposed asymptomatic, acutely, or mildly sick, and recovered cases	Has long incubation time and radiation risk	
	Enzyme-linked immune assays (EIA)	The enzyme-linked immunosorbent assay (ELISA) (Khanna et al., 2001)	Has high sensitivity, and it is a routine assay for protein detection, especially at significantly low levels (i.e., influenza virus infections)	Gives false-negative results during the window between the viral infection and the start of antibody production	
		Chemiluminescence immunoassay (CLIA) (Zhu et al., 2020)	-Same with ELISA<br>-It can be an automated	-Same with ELISA<br>-Requires complex equipment	
	Hemagglutination assay	adherence of red blood cells to the surface of the viruses infected cells (hemadsorption) plus visible agglutination of erythrocytes in specific patterns (Connor and Loeb 1983, Uhlendorff et al., 2009)	-It gives direct visualisation<br>-Can compare the relative concentrations of a virus between samples, such as those obtained from multiple infected hosts, or those collected sequentially from an individual host on different days or times.	-Similar to cell cultures<br>-Cannot accurately determine the number of virus particles present in a sample (i.e., virus particles/mL)	For virus isolation: inoculation of cell culture and subsequent demonstration of CPE, hemagglutinins, IFA, or hemadsorption (Dolskiy et al., 2020)
	Hemagglutination inhibition assay	Specific antibodies against viral antigens	-Gives direct visualisation.<br>-It is a fast and inexpensive method for vaccine, epidemiologic and antigenic cartography (Spackman and Sitaras 2020)	It is replaced by gene sequencing technology	

TABLE 1: Main diagnostic methods in virology

	Complement fixation test	reaction of the complement with an antigen-antibody complex	-Gives direct visualisation.	Its application is limited by the available suitable cross-reactive antigens to cover the existing virus serotypes	It is part of various protocols for respiratory viruses (Zhang et al. 2020)
New methods					
Nucleic acid amplification technique (NAAT)-based methods	Polymerase chain reaction (PCR) - the reference test	Viral proteins and nucleic acids detection for rapid known virus detection with known invariable portions of the genome	-It is fast, cheap, highly sensitive and specific detection and can be used for multiplex tests.<br>-rt RT-PCR assay provides faster quantitative analysis, with an equal or better sensitivity than cell culture methods (Van Elden et al., 2001)<br>-Has reduced contamination risk(Zhang et al., 2020)	-Requires specialised equipment and technical expertise about nucleotide sequence.<br>-Has long turnaround-time (multiple steps for sample preparation, difficult data processing).<br>-Gives false-positive results in persistent inactive infections with SARS-CoV-2, Epstein–Barr virus or adenovirus (Babiker et al., 2021) and difficult detection of unknown viruses and of viruses with highly variable genomes.	
	Loop-mediated amplification (LAMP) (Thi et al., 2020)		simple, rapid and cheap diagnostic tests performed without specialized equipment, in one single step, high sensitivity, possible POCT	may require additional sequence- specific detection using NGS or CRISPR	
	CRISPR like				LAMP + CRISPR
Next Generation Sequencing	sequencing methods for DNA and RNA sequencing (Bloom et al., 2020)	for new viruses, to identify mutations in the viral genome	can identify the viral nucleotide sequence, at high throughput data from multiple samples, and for possible large-scale testing	Developing technique	LAMP + NGS

TABLE 1: Main diagnostic methods in virology

Rapid antigen tests	lateral flow immunoassays,	for active infections	Rapid (10-30 minutes) test that can facilitate frequent decentralised testing at scale, with very few false positive results, and with high sensitivity for most infectious cases (Crozier et al. 2021)	-Gives false negative due to technical errors and during the 5-7 day incubation period and 1-2 days before symptom onset.(Lauer et al. 2020)<br>-The tests ‘performance falls when used by untrained staff or public (less when repeated)<br>- Infectious window is early and narrow (hard to find cases before they transmit infection).<br>- Does not quantify the level of virus material detected to reflect a level of infectiousness (Crozier et al., 2021)	In a low prevalence setting viruses can be detected by confirmatory PCR testing (https://apps.who.int/iris/handle/10665/334253)

Amarilla, A. A., N. Modhiran, Y. X. Setoh, N. Y. Peng, J. D. Sng, B. Liang, C. L. McMillan, M. E. Freney, S. T. Cheung and K. J. Chappell (2021). "An optimized high-throughput immuno-plaque assay for SARS-CoV-2." Frontiers in microbiology **12**.

Babiker, A., K. Immergluck, S. D. Stampfer, A. Rao, L. Bassit, M. Su, V. Nguyen, V. Stittleburg, J. M. Ingersoll and H. L. Bradley (2021). "Single-amplicon, multiplex real-time RT-PCR with tiled probes to detect SARS-CoV-2 spike mutations associated with variants of concern." Journal of clinical microbiology: JCM. 01446-01421.

Bloom, J. S., L. Sathe, C. Munugala, E. M. Jones, M. Gasperini, N. B. Lubock, F. Yarza, E. M. Thompson, K. M. Kovary and J. Park (2020). "Swab-Seq: A high-throughput platform for massively scaled up SARS-CoV-2 testing." MedRxiv.

Connor, E. M. and M. R. Loeb (1983). "A Hemadsorption Method for Detection of Colonies of Haemophilus influenzas Type b Expressing Fimbriae." Journal of Infectious Diseases **148**(5): 855-860.

Crozier, A., S. Rajan, I. Buchan and M. McKee (2021). "Put to the test: use of rapid testing technologies for covid-19." bmj **372**.

Dolskiy, A. A., I. V. Grishchenko and D. V. Yudkin (2020). "Cell Cultures for Virology: Usability, Advantages, and Prospects." International Journal of Molecular Sciences **21**(21): 7978.

Drosten, C., S. Günther, W. Preiser, S. Van Der Werf, H.-R. Brodt, S. Becker, H. Rabenau, M. Panning, L. Kolesnikova and R. A. Fouchier (2003). "Identification of a novel coronavirus in patients with severe acute respiratory syndrome." New England journal of medicine **348**(20): 1967-1976.

TABLE 1: Main diagnostic methods in virology

Goldsmith, C. S. and S. E. Miller (2009). "Modern uses of electron microscopy for detection of viruses." Clinical microbiology reviews **22**(4): 552-563.

Harcourt, J., A. Tamin, X. Lu, S. Kamili, S. K. Sakthivel, J. Murray, K. Queen, Y. Tao, C. R. Paden and J. Zhang (2020). "Severe acute respiratory syndrome coronavirus 2 from patient with coronavirus disease, United States." Emerging infectious diseases **26**(6): 1266.

Henrickson, K. J. (2004). "Advances in the laboratory diagnosis of viral respiratory disease." The Pediatric infectious disease journal **23**(1): S6-S10.

https://apps.who.int/iris/handle/10665/334253. Retrieved 08/12, 2021.

Johnson, K., P. Webb, J. Lange and F. Murphy (1977). "Isolation and partial characterisation of a new virus causing acute haemorrhagic fever in Zaire." Lancet: 569-571.

Khanna, M., P. Kumar, L. Chugh, A. Prasad and S. Chhabra (2001). "Evaluation of influenza virus detection by direct enzyme immunoassay (EIA) and conventional methods in asthmatic patients." The Journal of communicable diseases **33**(3): 163-169.

Kim, J.-H., S.-Y. Lee and S.-K. Lee (2021). "Development of novel lab-on-a-chip platform for high-throughput radioimmunoassay." Applied Radiation and Isotopes **168**: 109526.

Lauer, S. A., K. H. Grantz, Q. Bi, F. K. Jones, Q. Zheng, H. R. Meredith, A. S. Azman, N. G. Reich and J. Lessler (2020). "The incubation period of coronavirus disease 2019 (COVID-19) from publicly reported confirmed cases: estimation and application." Annals of internal medicine **172**(9): 577-582.

Leland, D. S. and C. C. Ginocchio (2007). "Role of cell culture for virus detection in the age of technology." Clinical microbiology reviews **20**(1): 49-78.

Lu, S., S. Lin, H. Zhang, L. Liang and S. Shen (2021). "Methods of Respiratory Virus Detection: Advances towards Point-of-Care for Early Intervention." Micromachines **12**(6): 697.

McClelland, R. D., T. N. Culp and D. J. Marchant (2021). "Imaging Flow Cytometry and Confocal Immunofluorescence Microscopy of Virus-Host Cell Interactions." Frontiers in Cellular and Infection Microbiology **11**.

Mendoza, E. J., K. Manguiat, H. Wood and M. Drebot (2020). "Two detailed plaque assay protocols for the quantification of infectious SARS-CoV-2." Current protocols in microbiology **57**(1): cpmc105.

Schulze-Horsel, J., Y. Genzel and U. Reichl (2008). "Flow cytometric monitoring of influenza A virus infection in MDCK cells during vaccine production." BMC biotechnology **8**(1): 1-12.

Soni, N., P. Pai, G. R. Krishna Kumar, V. Prasad, S. Dasgupta and B. Bhadra (2020). "A flow virometry process proposed for detection of SARS-CoV-2 and large-scale screening of COVID-19 cases." Future Virology **15**(8): 525-532.

Spackman, E. and I. Sitaras (2020). Hemagglutination inhibition assay. Animal Influenza Virus, Springer**:** 11-28.

Thi, V. L. D., K. Herbst, K. Boerner, M. Meurer, L. P. Kremer, D. Kirrmaier, A. Freistaedter, D. Papagiannidis, C. Galmozzi and M. L. Stanifer (2020). "A colorimetric RT-LAMP assay and LAMP-sequencing for detecting SARS-CoV-2 RNA in clinical samples." Science translational medicine **12**(556).

TABLE 1: Main diagnostic methods in virology

Uhlendorff, J., T. Matrosovich, H.-D. Klenk and M. Matrosovich (2009). "Functional significance of the hemadsorption activity of influenza virus neuraminidase and its alteration in pandemic viruses." Archives of virology **154**(6): 945-957.
Van Den Hoogen, B. G., D. Osterhaus and R. A. Fouchier (2004). "Clinical impact and diagnosis of human metapneumovirus infection." The Pediatric infectious disease journal **23**(1): S25-S32.
Van Elden, L., M. Nijhuis, P. Schipper, R. Schuurman and A. Van Loon (2001). "Simultaneous detection of influenza viruses A and B using real-time quantitative PCR." Journal of clinical microbiology **39**(1): 196-200.
Zhang, M., J. Ye, J.-s. He, F. Zhang, J. Ping, C. Qian and J. Wu (2020). "Visual detection for nucleic acid-based techniques as potential on-site detection methods. A review." Analytica chimica acta **1099**: 1-15.
Zhang, N., L. Wang, X. Deng, R. Liang, M. Su, C. He, L. Hu, Y. Su, J. Ren and F. Yu (2020). "Recent advances in the detection of respiratory virus infection in humans." Journal of medical virology **92**(4): 408-417.
Zhu, H., Z. Fohlerová, J. Pekárek, E. Basova and P. Neužil (2020). "Recent advances in lab-on-a-chip technologies for viral diagnosis." Biosensors and Bioelectronics **153**: 112041.
