# Supplementary material for: Advances in the Rapid Diagnostic of Viral Respiratory Tract Infections
Source: Front Cell Infect Microbiol. 2022 Feb 10;12:807253. doi: 10.3389/fcimb.2022.807253 (PMC8895598; doi:10.3389/fcimb.2022.807253)
Supplement: Supplementary file 2 [file Table_2.pdf]

TABLE 2: Specific diagnostic test devices in viral respiratory infections

Company	Kit	Virus	Method	Sample Type	Sensitivity	Specificity	Time	Highlights	References
**Commercially available respiratory virus detection tests/sample-in-answer-out detection systems**									
**Molecular Diagnostics**									
Cepheid (cepheid.com)	The GeneXpert Dx System	Influenza A/B, respiratory syncytial virus	multiplex rt RT-PCR	w/o pretreatment	Flu A: 100%<br>Flu B: 100%	Flu A: 100%<br>Flu B: 97.8%	7-30 min	automated sample-in-answer-out detection	(Baron and Persing 2019)
	GeneXpert® Xpress SARS-CoV-2	SARS-CoV-2	Real-time RT-qPCR	nasal swab, nasopharyngeal swab, aspirate specimens	100%	100%	45 min	meets the current urgent need for COVID-19 testing; sample-in-answer-out detection	(Loeffelholz et al., 2020, Zhen et al., 2020)
	GeneXpert® Xpress Flu/RSV	flu virus (A,B,RSV)	Real-time RT-PCR	nasal swab, nasopharyngeal swab specimens	Flu A: 98.1%<br>Flu B: 100%<br>RSV: 98.4%	Flu A: 98.1%<br>Flu B: 99.1%<br>RSV: 99.3%	20 min	Detects viral RNA, enabling better detection of seasonal mutations of the flu virus (A,b,RSV); sample-in-answer-out detection	(Ling et al., 2018)
Biofire(biofire dx.com)	The BioFire FilmArray® Respiratory (RP&RP2) Panels	VIRUSES:<br>Adenovirus; Coronavirus HKU1; Coronavirus NL63; Coronavirus 229E;Coronavirus OC43; Human Metapneumovirus; Human Rhinovirus/Enterovirus; Influenza A; Influenza A/H1; Influenza A/H3;Influenza A/H1-2009; Influenza B; Parainfluenza Virus 1/2/3/4; Respiratory Syncytial Virus<br>BACTERIA: Bordetella parapertussis; Bordetella pertussis; Chlamydia pneumoniae; Mycoplasma pneumoniae<br>* Available only on the BioFire Respiratory Panel 2	Real-time RT-PCR molecular test	nasopharyngeal swab in transport media	97.1%	99.3%	45 min	data indicated that the frozen storage did not significantly affect performance; sample-in-answer-out detection	(Leber et al., 2018)
	BioFire® Respiratory Panel 2.1-EZ (RP2.1-EZ)	VIRUSES:Adenovirus; Coronavirus;Human Metapneumovirus; Human Rhinovirus/ Enterovirus; Influenza A;Influenza A/H1; Influenza A/H3; Influenza A/H1-2009; Influenza B; Parainfluenza Virus; Respiratory<br>BACTERIA:<br>Bordetella pertussis; Chlamydophila pneumoniae; Mycoplasma pneumoniae	Real-time RT-PCR molecular test	nasopharyngeal swab	BAL: 96.2%<br>Sputum: 96.3%	BAL: 98.3%<br>Sputum:97.2 %	45 min	For use with the BioFire® FilmArray® 2.0 EZ Configuration System; sample-in-answer-out detection	BioFire® Respiratory Panel 2.1-EZ (RP2.1-EZ) ; (https://www.fda.gov/media/142696/download)
Luminex	Aries Flu A/B & RSV assay	18 respiratory virues and subtypes	MultiCode PCR	nasopharyngeal swab	98.6% (fluA), 93.3% (fluB) and 95.1% (RSV); in pediatric studies: 98.1% for influenza A virus, 98.0% for influenza B virus, and	100% for all targets; in pediatric studies: 98.6% - 99.8%	2 h	is a “sample to answer” automated	(Tang et al., 2016, Voermans et al., 2016)

TABLE 2: Specific diagnostic test devices in viral respiratory infections

					97.7% for RSV;				
Roche (diagnostics.roche.com)	Cobas Flu A/B & RSV	FluA, FluB, and RSV	RT-PCR	nasopharyngeal swabs	96.0 - 100.0% for all three viruses	99.3 - 100% for all three viruses	on-demand single-specimen: 21 min; batched 12-specimen workflows: 254 min	integrated nucleic acid extraction-independent devices for simultaneous detection and identification of the viral antigens; with no significant differences revealed among the Aries Flu A/B & RSV, the Xpert Xpress Flu/RSV, and the Cobas Flu A/B & RSV assays; sample-in-answer-out detection	(Ling et al., 2018, Jokela et al., 2020)
Mobidiag (mobidiag.com)	Novodiag® (Novodiag® COVID-19; Mobidiag Amplidiag® Covid-19 kit)	Novel coronavirus	RT-PCR	nasopharyngeal swabs	93.4%;	100%	~1 - 2,75h	fully automated Multiple RT-PCR and microarray detection of CarbaR+/Novel coronavirus/C. difficile; perform penicillin and myxomycetin resistance test; limited ability to detect low viral load samples	(Li et al., 2020)
**Immunology-Based Rapid Tests (RAT)**									
Abbott	SD Bioline Influenza Antigen Test®	Influenza virus type A&B and H1N1	antigen test	human nasal swab, throat swab, nasopharyngeal swab or nasal/nasopharyngeal aspirate	91.8%	98.9%	5 min	RAT for POCT to be complemented with PCR	SD BIOLINE Influenza Ag A/ B/ A(H1N1) Pandemic. Product Page.(https://www.alere.com/en/home/product-details/sd-bioline-influenza-ag-aba-pandemic.html)
Abbott	SD Bioline Influenza Ultra	Influenza virus type A&B	antigen test	nasopharyngeal swab, nasopharygeal aspirate	nasopharyngeal swab Flu A: 88.5% Flu B: 91.5% nasopharyngeal aspirate Flu A: 93.9% Flu B: 91.7%	nasopharyngeal swab Flu A: 97.4% Flu B: 97.4% nasopharyngeal aspirate Flu A: 97.7% Flu B: 97.7%	5–8 min		(Nelson et al., 2020)
Abbott	CLEARVIEW® EXACT INFLUENZA A&B	Influenza virus type A&B	chromatographic immunoassay antigen test	nasopharyngeal swab	Flu A: 81.7% Flu B: 88.6%	Flu A: 98.5% Flu B: 97.4%	15 min	insufficient sensitivity, prone to false negatives, test results to be combined with clinical practice for accurate diagnoses	(de la Tabla et al., 2010)
Abbott	Alere BINAXNOW® INFLUENZA A&B	Influenza virus type A&B	chromatographic immunoassay antigen test	nasopharyngeal swab, nasopharygeal aspirate	Flu A: 70–89% Flu B: 50–69%	Flu A: 90–99% Flu B: 94–100%	15 min	any age in the acute stage of the outbreak and in patients under 18 years of age	(Fuenzalida et al., 2010, Hazelton et al., 2015)

TABLE 2: Specific diagnostic test devices in viral respiratory infections

BioMedomics	COVID-19 IgM/IgG Rapid Test	SARS-CoV-2	serological antobody test	human serum	IgM: 86.7% IgG: 96.7% Combined: 100%	IgM: 95.0% IgG: 96.2% Combined: 92.5%	10-15 min	screen symptomatic or asymptomatic virus carriers; to verify the effectiveness of virus treatment.	Serology Test Evaluation Report BioMedomics, (https://www.accessdata.fda.gov/cdrh_docs/presentations/maf/maf3328-a001.pdf.)
Lucira Health (lucirahealth.com)	Lucira™ Covid-19 All-in-One Test Kit	SARS-CoV-2	LAMP technology	nasal swab	100% conformance rate compared to highly sensitive detection methods		30 min	the first home self-test kit FDA emergency authorized (November 2020) for sample-in-answer-out detection	FDA Authorizes First COVID-19 Test for Self-Testing at Home.(https://www.fda.gov/news-events/press-announcements/coronavirus-covid-19-update-fda-authorizes-first-covid-19-test-self-testing-home)
**Advances in Respiratory Virus Testing for Rapid Diagnosis (Research)**									
**Rapid Diagnsotic Test**									
	RT-LAMP diagnostic platfoms	influenza viruses (H5N1, H5N6, H1N1, H3N2 and H7N9), MERS	colorimetric multiplex reverse transcription LAMP (RT-LAMP)		Influenza: 100%; MERS higher than RT-LAMP Assay	high; no cross-reactivity with any of SARS-CoV, HKU4, HKU1, OC43 or 229E	1h	for POC testing	(Huang et al., 2018, Ahn et al., 2019)
**Immunoassay Paper-Based Devices/Technology**									
	Lateral flow test stripes	H1N1 and HAdV	surface-enhanced Raman scattering (SERS) spectroscopy	human serum	detection limit for H1N1: 50pfu/mL; HAdV 10 pfu/mL (2000-fold lower than the standard gold strip)	not specified	Rapid test	potential to detect coronavirus, in complex specimens.	(Wang et al., 2019)
	Lateral flow test stripes	SARS-CoV-2	surface-enhanced Raman scattering (SERS) spectroscopy	venous blood samples and finger blood samples	IgM, IgG combined: 88.66%	IgM, IgG combined: 90.63%	Rapid test	test symptomatic and asymptomatic SARS-CoV-2 carriers at the POC	(Li et al., 2020)
**Biosensors for Respiratory Virus Detection**									
	mini 3D-printed readout biosensor platform	avian influenza A (H7N9) virus	quantum dot (QD)-based immunochromatographic strips		detection limit of 0.0268 HAU	was 98% conformance rate compared to real-time PCR	15 min	assessment of the result by the naked eye	(Xiao et al., 2019)
	electrochemical biosensor	influenza virus A (H9N2)	electrochemical biosensor	pharyngeal swab	detection limit of 10^{-8} U/mL	NA	30 -60 min	improved properties of the biosensor for POCT	(Anik et al., 2018)

TABLE 2: Specific diagnostic test devices in viral respiratory infections

	electrochemical immunosensor	MERS-CoV	carbon electrode dielectrophoresis (DEP) array		0.001 ng/mL; improved to 1.0 pg/mL	NA	20 min	potential for other respiratory viruses' detection; immunological biosensors cannot be used for accurate detection in the early stages of viral infection;	(Layqah and Eissa, 2019)
	impedimetric immunobiosensor	influenza A viruses	electrochemical probe	serum viral antigen	detection limit: 0.79 fM; linear range: 0.18 f. - 0.18 nM	NA	NA		(Dunajová et al., 2020)
	arch-shaped multiple-target sensors platform	MERSCoV, HCoV, EBOV and ZIKV	oligonucleotides amplification	serum viral antigen	high	high	20 min	detect the viruses in 20 clinical specimens	(Dunajová et al., 2020)
**Microfluidic Device for Respiratory Virus Detection**									
	RNA assay chip	influenza A and B virus and human adenoviruses	PCR+ colorimetry		96% (CI: 78.1 - 99.9)	100% (CI:94.9 - 100.0)	<1h		(Wang et al., 2018)
	RNA assay chip	SARS-CoV, MERS-CoV, SARS-CoV-2, HCoV (HCoV-OC43, HCoV-229E, HCoV-HKU1, HCoV-NL63)	LAMP		100%		40 min	portable microfluidic system for simultaneous deetction of coronaviruses	(Xiong et al., 2020)
	RTisochip™ -W	H7N9, CA16, RSV, HPIV1, HPIV2, HPIV3, HPIV4, HCoV-229E/NL63, HMPV, H1N1, H3N2, influenza B, EV71, HCoV-OC43/HKU1, CA6, HRV, SARS-CoV-2	multi-index nucleic acid isothermal amplification	pharyngeal swab	detection limit : <50 copies/ uL (equivalent to RT-PCR)	high; 98.5% coincidence rate with the RT-PCR results	90 min	multi-index nucleic acid isothermal amplification analyzer with a centrifugal microfluidic chip	(Xing et al., 2020)
**Smartphone-Based Detection Technologies**									
	3D nanostructures platform	avian influenza virus	ZnO nanorod-based colorimetric immunoassay	serum viral antigen	LOD: 8x10e3 EID50/mL - 2.7 x10e4 EID50/mL	NA	1.5 h	Sensitiviy higher than the conventional fluorescence-based ELISA assay	(Xia et al., 2019)
	ddPCR platform	RNA load	droplet digital PCR fluorescence reading	spiked saliva	LODs : 3.8 copies/20 uL of sample	dynamic range of 4–100 copies	1.5 h	RNA extraction may not be necessary	(Chen et al., 2021)
	SERS imaging biosensor	influenza A/H1N1 virus	Surface-enhanced Raman scattering (SERS)		LOD: 97 PFU mL−1;	NA	20 min	three orders of magnitude more sensitive than ELISA	(Chen et al., 2020)
	droplet-based digital LAMP	ctDNA	digital IFAST(immiscible phase filtration assisted by surface tension)-LAMP	spiked plasma	recovery rate 75%	NA	1h	complicated manual operations	(Hu et al., 2019)

TABLE 2: Specific diagnostic test devices in viral respiratory infections

	IddPCR microdevice	ctDNA	PCR	spiked plasma	for EGFR Gene T790 M mutation: 1% mutant from 2 mL of plasma sample	NA	~1h	a fully integrated digital droplet PCR microdevice liquid biopsy	(Geng et al., 2020)

Ahn, S. J., Y. H. Baek, K. K. S. Lloren, W.-S. Choi, J. H. Jeong, K. J. C. Antigua, H.-i. Kwon, S.-J. Park, E.-H. Kim and Y.-i. Kim (2019). "Rapid and simple colorimetric detection of multiple influenza viruses infecting humans using a reverse transcriptional loop-mediated isothermal amplification (RT-LAMP) diagnostic platform." BMC infectious diseases **19**(1): 1-12.

Anik, Ü., Y. Tepeli, M. Sayhi, J. Nsiri and M. F. Diouani (2018). "Towards the electrochemical diagnostic of influenza virus: Development of a graphene–Au hybrid nanocomposite modified influenza virus biosensor based on neuraminidase activity." Analyst **143**(1): 150-156.

Baron, E. J. and D. H. Persing (2019). "Invited Product Profile–GeneXpert Xpress System for Respiratory Testing: State-of-the-Art Molecular Testing for Any Health Care Setting." Point of Care **18**(2): 66-71.

Chen, H., S.-G. Park, N. Choi, J.-I. Moon, H. Dang, A. Das, S. Lee, D.-G. Kim, L. Chen and J. Choo (2020). "SERS imaging-based aptasensor for ultrasensitive and reproducible detection of influenza virus A." Biosensors and Bioelectronics **167**: 112496.

Chen, L., V. Yadav, C. Zhang, X. Huo, C. Wang, S. Senapati and H.-C. Chang (2021). "Elliptical Pipette Generated Large Microdroplets for POC Visual ddPCR Quantification of Low Viral Load." Analytical Chemistry **93**(16): 6456-6462.

de la Tabla, V. O., P. Antequera, M. Masiá, P. Ros, C. Martin, G. Gazquez, F. Buñuel, V. Sánchez, C. Robledano and F. Gutiérrez (2010). "Clinical evaluation of rapid point-of-care testing for detection of novel influenza A (H1N1) virus in a population-based study in Spain." Clinical microbiology and infection **16**(9): 1358-1361.

Dunajová, A. A., M. Gál, K. Tomčíková, R. Sokolová, V. Kolivoška, E. Vaněčková, F. Kielar, F. Kostolanský, E. Varečková and M. Naumowicz (2020). "Ultrasensitive impedimetric imunosensor for influenza A detection." Journal of Electroanalytical Chemistry **858**: 113813.

Fuenzalida, L., S. Blanco, C. Prat, M. Vivancos, M. Dominguez, J. M. Mòdol, C. Rodrigo and V. Ausina (2010). "Utility of the rapid antigen detection BinaxNOW Influenza A&B test for detection of novel influenza A (H1N1) virus." Clinical microbiology and infection **16**(10): 1574-1576.

Geng, Z., S. Li, L. Zhu, Z. Cheng, M. Jin, B. Liu, Y. Guo and P. Liu (2020). "“Sample-to-Answer” Detection of Rare ctDNA Mutation from 2 mL Plasma with a Fully Integrated DNA Extraction and Digital Droplet PCR Microdevice for Liquid Biopsy." Analytical chemistry **92**(10): 7240-7248.

Hazelton, B., T. Gray, J. Ho, V. M. Ratnamohan, D. E. Dwyer and J. Kok (2015). "Detection of influenza A and B with the Alere™ i Influenza A & B: a novel isothermal nucleic acid amplification assay." Influenza and other respiratory viruses **9**(3): 151-154.

https://www.accessdata.fda.gov/cdrh_docs/presentations/maf/maf3328-a001.pdf.

https://www.alere.com/en/home/product-details/sd-bioline-influenza-ag-aba-pandemic.html.

https://www.fda.gov/media/142696/download.

https://www.fda.gov/news-events/press-announcements/coronavirus-covid-19-update-fda-authorizes-first-covid-19-test-self-testing-home.

Hu, F., J. Li, Z. Zhang, M. Li, S. Zhao, Z. Li and N. Peng (2019). "Smartphone-based droplet digital LAMP device with rapid nucleic acid isolation for highly sensitive point-of-care detection." Analytical chemistry **92**(2): 2258-2265.

TABLE 2: Specific diagnostic test devices in viral respiratory infections

Huang, P., H. Wang, Z. Cao, H. Jin, H. Chi, J. Zhao, B. Yu, F. Yan, X. Hu and F. Wu (2018). "A rapid and specific assay for the detection of MERS-CoV." Frontiers in microbiology **9**: 1101.

Jokela, P., A. E. Jääskeläinen, H. Jarva, T. Holma, M. J. Ahava, L. Mannonen, M. Lappalainen, S. Kurkela and R. Loginov (2020). "SARS-CoV-2 sample-to-answer nucleic acid testing in a tertiary care emergency department: evaluation and utility." Journal of Clinical Virology **131**: 104614.

Layqah, L. A. and S. Eissa (2019). "An electrochemical immunosensor for the corona virus associated with the Middle East respiratory syndrome using an array of gold nanoparticle-modified carbon electrodes." Microchimica Acta **186**(4): 1-10.

Leber, A. L., K. Everhart, J. A. Daly, A. Hopper, A. Harrington, P. Schreckenberger, K. McKinley, M. Jones, K. Holmberg and B. Kensinger (2018). "Multicenter evaluation of BioFire FilmArray Respiratory Panel 2 for detection of viruses and bacteria in nasopharyngeal swab samples." Journal of clinical microbiology **56**(6): e01945-01917.

Li, Z., Y. Bai, M. You, J. Hu, C. Yao, L. Cao and F. Xu (2020). "Fully integrated microfluidic devices for qualitative, quantitative and digital nucleic acids testing at point of care." Biosensors and Bioelectronics: 112952.

Li, Z., Y. Yi, X. Luo, N. Xiong, Y. Liu, S. Li, R. Sun, Y. Wang, B. Hu and W. Chen (2020). "Development and clinical application of a rapid IgM-IgG combined antibody test for SARS-CoV-2 infection diagnosis." Journal of medical virology **92**(9): 1518-1524.

Ling, L., S. E. Kaplan, J. C. Lopez, J. Stiles, X. Lu and Y.-W. Tang (2018). "Parallel validation of three molecular devices for simultaneous detection and identification of influenza A and B and respiratory syncytial viruses." Journal of clinical microbiology **56**(3): e01691-01617.

Loeffelholz, M. J., D. Alland, S. M. Butler-Wu, U. Pandey, C. F. Perno, A. Nava, K. C. Carroll, H. Mostafa, E. Davies and A. McEwan (2020). "Multicenter evaluation of the cepheid Xpert Xpress SARS-CoV-2 test." Journal of clinical microbiology **58**(8): e00926-00920.

Nelson, P. P., B. A. Rath, P. C. Fragkou, E. Antalis, S. Tsiodras and C. Skevaki (2020). "Current and future point-of-care tests for emerging and new respiratory viruses and future perspectives." Frontiers in cellular and infection microbiology **10**: 181.

Tang, Y.-W., S. Gonsalves, J. Y. Sun, J. Stiles, K. A. Gilhuley, A. Mikhlina, S. A. Dunbar, N. E. Babady and H. Zhang (2016). "Clinical evaluation of the Luminex NxTAG respiratory pathogen panel." Journal of clinical microbiology **54**(7): 1912-1914.

Voermans, J. J., S. Seven-Deniz, P. Fraaij, A. A. van der Eijk, M. Koopmans and S. D. Pas (2016). "Performance evaluation of a rapid molecular diagnostic, MultiCode based, sample-to-answer assay for the simultaneous detection of influenza A, B and respiratory syncytial viruses." Journal of Clinical Virology **85**: 65-70.

Wang, C., C. Wang, X. Wang, K. Wang, Y. Zhu, Z. Rong, W. Wang, R. Xiao and S. Wang (2019). "Magnetic SERS strip for sensitive and simultaneous detection of respiratory viruses." ACS applied materials & interfaces **11**(21): 19495-19505.

Wang, R., R. Zhao, Y. Li, W. Kong, X. Guo, Y. Yang, F. Wu, W. Liu, H. Song and R. Hao (2018). "Rapid detection of multiple respiratory viruses based on microfluidic isothermal amplification and a real-time colorimetric method." Lab on a Chip **18**(22): 3507-3515.

Xia, Y., Y. Chen, Y. Tang, G. Cheng, X. Yu, H. He, G. Cao, H. Lu, Z. Liu and S.-Y. Zheng (2019). "Smartphone-based point-of-care microfluidic platform fabricated with a ZnO nanorod template for colorimetric virus detection." ACS sensors **4**(12): 3298-3307.

Xiao, M., L. Huang, X. Dong, K. Xie, H. Shen, C. Huang, W. Xiao, M. Jin and Y. Tang (2019). "Integration of a 3D-printed read-out platform with a quantum dot-based immunoassay for detection of the avian influenza A (H7N9) virus." Analyst **144**(8): 2594-2603.

Xing, W., Y. Liu, H. Wang, S. Li, Y. Lin, L. Chen, Y. Zhao, S. Chao, X. Huang and S. Ge (2020). "A high-throughput, multi-index isothermal amplification platform for rapid detection of 19 types of common respiratory viruses including SARS-CoV-2." Engineering.

TABLE 2: Specific diagnostic test devices in viral respiratory infections

Xiong, H., X. Ye, Y. Li, L. Wang, J. Zhang, X. Fang and J. Kong (2020). "Rapid differential diagnosis of seven human respiratory coronaviruses based on centrifugal microfluidic nucleic acid assay." Analytical chemistry **92**(21): 14297-14302.
Zhen, W., E. Smith, R. Manji, D. Schron and G. J. Berry (2020). "Clinical evaluation of three sample-to-answer platforms for detection of SARS-CoV-2." Journal of clinical microbiology **58**(8): e00783-00720.
