# Supplementary material for: Advances in the Rapid Diagnostic of Viral Respiratory Tract Infections
Source: Front Cell Infect Microbiol. 2022 Feb 10;12:807253. doi: 10.3389/fcimb.2022.807253 (PMC8895598; doi:10.3389/fcimb.2022.807253)
Supplement: Supplementary file 3 [file Table_3.pdf]

Table 3: IoT approaches in COVID-19 management

IoT type	Example	Description	References
**Smartphone applications**	DetectaChem (USA)	Low cost COVID-19 testing using a kit connected to a smartphone application	Nasajpour et al., 2020
	Stop Corona (Croatia)	Provides predictive heatmaps based on the disease spots	United against coronavirus! Stopcorona App. (2020) https://stopcorona.app/
	eRouska (smart quarantine) -Czech Republic	Captures physical contacts between user and people	https://erouska.cz/en.
	StayHomeSafe (Hong Kong)	Monitoring of arrivals at the airport with use of smartphone application and a wristband	Kondylakis, 2020<br>Nasajpour et al., 2020
	COVID-19 Intelligent Diagnosis and Treatment Assistant Program (nCapp)-China	an automated diagnosis system – it can automatically generate a diagnosis report based on requested data and questionnaires submitted by patients	Bai et al. (2020)
	Hamagen (Israel)<br>Coalition (USA)<br>BeAware Bahrain	Contact tracing	Nasajpour et al., 2020
**Wearables**	IoT-Q-Band	Tracker wristband-Tracking of quarantined cases in case of absconding	Singh et al, 2020
	Proximity Trace	Hardhat TraceTag - monitoring workers for social distancing	https://bit.ly/2B60rF2<br>Nasajpour et al., 2020
	EasyBand	Pact wristband -monitor for social distance	Tripathy, 2020
	Smart glasses	Temperature monitoring	Nasajpour et al., 2020
	Smart helmet	Temperature monitoring	Nasajpour et al., 2020
	Smart thermometers	Temperature monitoring	Nasajpour et al., 2020
**Drones**	Thermal imaging drone (Pandemic Drone)	Temperature monitoring in a crowd	Nasajpour et al., 2020
	Announcement drone	Broadcasting information about COVID-19	Nasajpour et al., 2020
	Disinfectant drone (DJI)	Sterilisation of contaminated areas	Nasajpour et al., 2020
	Surveillance drone (MicroMultiCopter)	Monitoring of social distancing	Nasajpour et al., 2020
	Multipurpose drone (Corona Combat)	Disinfection, monitoring od temperature, broadcasting of information	Nasajpour et al., 2020
**Robots**	Telerobots- DaVinci surgical robots	Reduction of the infection risk for medical staff	Khan, 2020
	Autonomous robots -Spot robot	Disinfection, collection of swabs, checking and treatment of patients<br>Prevention of the risk of infection of the medical staff	Nasajpour et al., 2020<br>Khan, 2020

Table 3: IoT approaches in COVID-19 management

	Collaborative robots – eXtreme Disinfection Robot, Asimov Robotics	Complementary to the healthcare staff- disinfection	Yang, 2020
**IoT buttons small, programmable button connected to the cloud**	Wanda QuickTouch	Alerts the authorities or families	Nasajpour et al., 2020
	Sefucy	Alerts healthcare providers in case of an emergency	Nasajpour et al., 2020

Swayamsiddha, S, Mohanty, C (2020). "Application of cognitive Internet of Medical Things for COVID-19 pandemic". Diabetes Metab Syndr. **14**(5):911-915. doi:10.1016/j.dsx.2020.06.014

Yang, G., Lv, H., Zhang, Z., Yang, L., Deng, J., You, S., Du, J., Yang, H (2020). "Keep healthcare workers safe: application of teleoperated robot in isolation ward for COVID-19 prevention and control". Chin J Mech Eng **33**(1):1–4

Bai, L., Yang, D., Wang, X., Tong, L., Zhu, X., Zhong, N., Bai, C., Powell, C.A., Chen, R., Zhou, J., et al. (2020) "Chinese experts' consensus on the internet of things-aided diagnosis and treatment of coronavirus disease 2019 (COVID-19) ". Clinical eHealth **3**:7–15

Nasajpour, M., Pouriyeh, S., Parizi, RM., Dorodchi, M., Valero, M., Arabnia, HR. (2020) "Internet of Things for Current COVID-19 and Future Pandemics: an Exploratory Study". J Healthc Inform Res. **12**:1-40. doi: 10.1007/s41666-020-00080-6.

Singh, V.K., Chandna, H., Kumar, A., Kumar, S., Upadhyay, N., Utkarsh, K (2020). "IoT-Q-Band: a low cost internet of things based wearable band to detect and track absconding COVID-19 quarantine subjects". EAI Endorsed Transactions on Internet of Things, **20**(21):163997, DOI:10.4108/eai.13-7-2018.163997

Tripathy, A.K., Mohapatra, A.G., Mohanty, S.P., Kougianos, E., Joshi, A.M., Das, G (2020) "Easyband: a wearable for safety-aware mobility during pandemic outbreak". IEEE Consumer Electronics Magazine

Ye, Q., Zhou, J., Wu, H (2020). "Using Information Technology to Manage the COVID-19 Pandemic: Development of a Technical Framework Based on Practical Experience in China". JMIR Med Inform. **8**(6):e19515. doi: 10.2196/19515. PMID: 32479411;

Khan, Z.H., Siddique, A., Lee, C.W. "Robotics Utilization for Healthcare Digitization in Global COVID-19 Management" (2020). Int J Environ Res Public Health. **17**(11):3819. doi:10.3390/ijerph17113819
